# Metabolic control of PPAR activity by aldehyde dehydrogenase regulates invasive cell behavior and predicts survival in hepatocellular and renal clear cell carcinoma

**DOI:** 10.1186/s12885-018-5061-7

**Published:** 2018-11-28

**Authors:** Diana Andrejeva, Jan-Michael Kugler, Hung Thanh Nguyen, Anders Malmendal, Mette Lind Holm, Birgitte Groenkaer Toft, Anand C. Loya, Stephen M. Cohen

**Affiliations:** 1 Department of Cellular and Molecular Medicine, University of Copenhagen, Blegdamsvej 3, DK-2200 Copenhagen N, Denmark; 2 Department of Urology, Rigshospitalet, Blegdamsvej 9, DK-2100 Copenhagen Ø, Denmark; 3 Department of Pathology, Rigshospitalet, Blegdamsvej 9, DK-2100 Copenhagen Ø, Denmark

## Abstract

**Background:**

Changes in cellular metabolism are now recognized as potential drivers of cancer development, rather than as secondary consequences of disease. Here, we explore the mechanism by which metabolic changes dependent on aldehyde dehydrogenase impact cancer development.

**Methods:**

ALDH7A1 was identified as a potential cancer gene using a Drosophila in vivo metastasis model. The role of the human ortholog was examined using RNA interference in cell-based assays of cell migration and invasion. 1H-NMR metabolite profiling was used to identify metabolic changes in ALDH7A1-depleted cells. Publically available cancer gene expression data was interrogated to identify a gene-expression signature associated with depletion of ALDH7A1. Computational pathway and gene set enrichment analysis was used to identify signaling pathways and cellular processes that were correlated with reduced ALDH7A1 expression in cancer. A variety of statistical tests used to evaluate these analyses are described in detail in the methods section. Immunohistochemistry was used to assess ALDH7A1 expression in tissue samples from cancer patients.

**Results:**

Depletion of ALDH7A1 increased cellular migration and invasiveness in vitro*.* Depletion of ALDH7A1 led to reduced levels of metabolites identified as ligands for Peroxisome proliferator-activated receptor (PPARα). Analysis of publically available cancer gene expression data revealed that ALDH7A1 mRNA levels were reduced in many human cancers, and that this correlated with poor survival in kidney and liver cancer patients. Using pathway and gene set enrichment analysis, we establish a correlation between low ALDH7A1 levels, reduced PPAR signaling and reduced patient survival. Metabolic profiling showed that endogenous PPARα ligands were reduced in ALDH7A1-depleted cells. ALDH7A1-depletion led to reduced PPAR transcriptional activity. Treatment with a PPARα agonist restored normal cellular behavior. Low ALDH7A1 protein levels correlated with poor clinical outcome in hepatocellular and renal clear cell carcinoma patients.

**Conclusions:**

We provide evidence that low ALDH7A1 expression is a useful prognostic marker of poor clinical outcome for hepatocellular and renal clear cell carcinomas and hypothesize that patients with low ALDH7A1 might benefit from therapeutic approaches addressing PPARα activity.

**Electronic supplementary material:**

The online version of this article (10.1186/s12885-018-5061-7) contains supplementary material, which is available to authorized users.

## Background

A growing body of evidence links changes in metabolism to cancer [1, 2]. In addition to the well-known shift of cancer cells to aerobic glycolysis, mutations or changes in the expression of metabolic enzymes have been identified as potential cancer drivers. Mutations and/or altered expression of metabolic enzymes such as succinate dehydrogenase, pyruvate kinase and isocitrate dehydrogenase are linked to tumor initiation, development and drug resistance [3–6]. Changes in metabolite levels can affect expression profiles, epigenetic marks and chromatin organization in cancer, with resulting changes in cellular phenotypes, metastatic potential, as well as on the tumor microenvironment [7].

The human ALDH family comprises 19 enzymes that catalyze NAD(P)+ dependent oxidation of aldehydes to their corresponding carboxylic acids and NAD(P)H [8]. Notably, ALDH1 is thought to be oncogenic in breast cancer. Cells with high ALDH1 activity have been linked to poor outcome in some cancers [9, 10], albeit not in others [11, 12]. Evidence of the roles of other ALDH isoforms in cancer remains equivocal.

In this study, we provide evidence for a role of ALDH isoform 7A1 (ALDH7A1) in human cancer, and link this to regulation of PPAR activity. PPARs (Peroxisome proliferator-activated receptors) are ligand-activated transcription factors, regulated by cellular metabolites [13, 14]. Metabolite-regulated control of PPAR activity contributes to cellular homeostasis through feedback regulation on the expression on enzymes involved in glucose, amino acid and lipid metabolism [15]. Metabolic profiling showed that ALDH7A1-depletion reduced the levels of metabolites that serve as activating ligands for PPARs. Analysis of cancer RNAseq data from TCGA showed that low ALDH7A1 mRNA levels correlate with a low PPAR activity signature, and with poor survival prognosis in patients with hepatocellular carcinoma and renal clear cell carcinoma. Importantly, the cellular phenotypes associated with ALDH7A1-depletion, increased migration and invasiveness, were corrected by restoring PPAR activity. We hypothesize that metabolic changes resulting from low ALDH7A1 expression may be linked to clinical outcome through their effects on PPAR activity. PPARs are pharmaceutical targets for metabolic disorders including diabetes, dyslipidemia, obesity, chronic inflammation and atherosclerosis [16, 17]. Immunohistochemical staining of clinical samples suggests that low ALDH7A1 expression may be a useful prognostic marker of poor clinical outcome for hepatocellular and renal clear cell carcinomas. Our findings suggest a route to identifying cancer patients who might benefit from PPAR agonist therapy.

## Methods

### Cells

Primary BJ cells were originally obtained from ATCC (Cat# ATCC® CRL-2522™). hTert-expressing BJ cells were engineered to express p53 and p16 shRNAs (4F3). These genetic modifications enable cells to migrate and invade well in migration and invasion assays. Cells were expanded to passage 5, and frozen. All subsequent experiments were performed using this parental polyclonal 4F3 cell line. BJ cells were tested for mycoplasma every 6 months and examined for consistent phenotype and behavior on an ongoing basis. Information on the other cell lines used in this study is provided in Additional file 1: Figure S7.

### Viral transduction

Lentivirus particles were produced by calcium phosphate transfection of 293 T cells and harvested after 24 h–48 h using standard procedures. One to two passages after thawing, BJ-4F3, HUH7, CAKI2 cells were transduced with control shRNAs (Sigma: SHC001 as empty vector, SHC002 as non-targeting shRNA control) or ALDH7A1-specific shRNAs (Sigma: TRCN0000028424 (sh1) and TRCN0000028447 (sh2)) for 24 h, allowed to recover for 24 h, and placed under puromycin selection (2 μg/ml) for 6 days. Experiments were performed within the next 10 passages. All experiments were performed at least 3 times with independently transduced cells. Knockdown efficiency was assessed by quantitative RT-PCR (qPCR) (forward primer: CATGGCGTGAGTGAAGGAC, reverse primer: CAGGGCAATAGGTCGTAATAACC), and/or by immunoblotting of cell extracts using rabbit anti-ALDH7A1 (Sigma: HPA023296).

### Quantitative qPCR

Total RNA was isolated with the “RNeasy Plus Mini Kit” following the manufacturer’s instructions. After DNase treatment (RQ1 RNase-Free DNase; Promega) a cDNA was synthesed using a SuperScript™ III First-Strand Synthesis System (Invitrogen) using 0.5–1 μg of total RNA. qPCR was carried out on a QuantStudio 6 Flex Real-Time PCR System (Applied Biosystems) using HOT FIREPol® EvaGreen® qPCR Mix Plus (ROX) (SOLIS BIODYNE). Total RNA from each sample was normalized to β-ACTIN, KIF and TBP for the BJ and HUH7 cell line or KIF in the case of CAKI2 cell line. Significance was determined using the Mann–Whitney U test after adjusting for False Discovery Rate. The following primers were used: CYP27A1 (forward primer: GGTGCTTTACAAGGCCAAGTA, reverse primer: TCCCGGTGCTCCTTCCATAG), FABP3 (forward primer: TGGAGTTCGATGAGACAACAGC, reverse primer: CTCTTGCCCGTCCCATTTCTG), ACSL1 (forward primer: CTTATGGGCTTCGGAGCTTTT, reverse primer: CAAGTAGTGCGGATCTTCGTG), CPT2 (forward primer: CTGGAGCCAGAAGTGTTCCAC, reverse primer: AGGCACAAAGCGTATGAGTCT), ACOX1 (forward primer: ACTCGCAGCCAGCGTTATG, reverse primer: AGGGTCAGCGATGCCAAAC), FADS2 (forward primer: AATCAGCAGGGGTTTCAAGA, reverse primer: GGCACTACGCTGGAGAAGAT), APOA1 (forward primer: TTGCTGAAGGTGGAGGTCAC, reverse primer: TGGATGTGCTCAAAGACAGC), β-ACTIN (forward primer: GATGCGTAGCATTTGCTGCATGG, reverse primer: TGAGGCTAGCATGAGGTGTGTG), TBP (forward primer: CGCCGAATATAATCCCAAGC, reverse primer: TCCTGTGCACACCATTTTCC), KIF (forward primer: TTGCCTCCTTTGGCAACATTCG, reverse primer: ACACAGCACCAATACCCATGATAC).

BJ cells were treated with PPARα agonist (Ciprofibrate) or DMSO as a control. Cells were collected for RNA extraction and qPCR as described above. β-ACTIN was used as normalization control. Friedman rank sum test with pairwise post-hoc test for multiple comparisons with “holms” adjustment was used to calculate *p*-values between groups with and without Ciprofibrate treatment.

### Cell culture

Unless specifically mentioned, all cell lines were cultured in high glucose DMEM (Dulbecco’s Modified Eagle Medium; Sigma-Aldrich) with 10% Fetal Calf Serum (Sigma) and 1% Penicillin-Streptomycin (Sigma), 1% GlutaMAX™-I (Gibco) and 1% pyruvate. Cells were cultured at 37 °C in a humidified environment containing 5% CO_2_.

### Phenotypic assays

Cell proliferation assays were performed by plating BJ-4F3 cells at a density of 2 × 10^4^ cells/cm^2^ in triplicate wells. Cells were grown for 3, 24, 48, 72 or 96 h and then fixed with 4% formaldehyde (Sigma-Aldrich). The number of DAPI-stained nuclei was counted in representative images of each well, at each time point. Data are presented as the fold change in cell number over time (± standard error of the mean).

Wound healing assays were performed by plating transduced BJ-4F3 cells (at 4 × 10^4^ cells/cm^2^), HUH7 cells (7 × 10^4^ cells/cm^2^) and CAKI2 cells (5 × 10^4^ cells/cm^2^). Cells were allowed to form a monolayer for 24 h. A stripe was cleared by dragging a pipet tip across the surface of the plate, and the culture medium was changed to wash away floating cells. The initial state was recorded by taking 2–4 images at defined places (4x magnification; *t* = 0). Cells were allowed to migrate for 24 h and images were taken of the same regions. The area devoid of cells was measured and the average migrated distance calculated.

Invasive migration assays (transwell) were performed using transduced BJ-4F3 cells, that had been serum-starved for 24 h. Matrigel invasion chambers with 8.0 μm Polyethylene Terephthalate membranes were used according to manufacturer’s protocol (Fisher Scientific, #11553570). Complete DMEM supplemented with 20% FCS was used as attractant at the bottom of the well. 5 × 10^4^ cells were seeded on top of the Matrigel in serum-free complete DMEM. After 24 h the chamber was washed once with PBS and cells were fixed with 4% formaldehyde. Nuclei were stained with DAPI and counted to determine the number of cells in the upper invasion chamber. The inside of the chamber was then cleared and the cells that had migrated through the gel were counted. Ten pictures were taken per chamber at 10x magnification. The total number of cells in the invasion chamber was used for normalization. Cell number and migrated distance were measured with ImageJ Fiji software.

Cells were treated with Ciprofibrate, GW501516, or Rosiglitazone at the concentration indicated in the figures at *t* = 0 of the scratch assay, and at seeding time in the invasion assays (both chambers).

### TCGA data

The publicly available RNA sequencing data and clinical information was downloaded from The Broad Institute TCGA GDAC Firehose on 08.08.2016, release version 2016_01_28. (https://portal.gdc.cancer.gov/) (http://firebrowse.org/). Normalized (RNA-seq expectation by maximization) data was used. Patient follow up information was downloaded from https://portal.gdc.cancer.gov/ using R package “TCGAbiolinks” on 01.12.2016 [18]. For Additional file 1: Figure S4, RNA sequencing data were downloaded from TCGA (version 8.0) (https://portal.gdc.cancer.gov/), using “TCGAbiolinks”. Upper quartile normalized fragments per Kilobase of transcript per million mapped reads data was used. Reverse phase protein array data were downloaded from http://tcpaportal.org/tcpa/ on 12.10.2017.

### Expression, correlation and survival analysis

Statistical analysis was performed using R Software. For mRNA expression, significance was determined using the Mann–Whitney U test. To calculate comparison between multiple groups, pairwise Wilcoxon test with Bonferroni correction for multiple testing was applied. For overall survival analysis, cancer patients were divided into three equally sized groups based on ALDH7A1 mRNA expression levels (low, middle, high). Cox proportion hazard regression models were used to calculate *p*-values between groups.

For Additional file 1: Figure S4 patients were divided into two equally sized groups based on ALDH7A1 expression, EGFR expression and sum of scaled and centered relative protein levels EGFR_pY1068 (CST; 2234), EGFR_pY1173 (Abcam; ab32578). Hazard Ratio for low ALDH7A1 expression and associated *p*-value was calculated in EGFR low and high groups separately. For correlation analysis Spearman coefficients and corresponding *p*-values were calculated between ALDH7A1 RNA expression and EGFR RNA expression, and the sum of scaled and centered phosphorylated EGFR protein levels.

### Pathway and gene set enrichment analysis

The R/bioconductor package limma [19] was used to identify genes differentially expressed between the top and bottom third ALDH7A1 expression groups. Data were filtered using RSEM > 10 in at least in 33% of samples to reduce noise from low expressed transcripts. Genes with log_2_-fold change +/− 0.4 with adjusted *p*-value threshold < 0.05 were defined as differentially expressed between groups. All genes not eliminated by filtering were used to define the “gene universe” for pathway enrichment analysis.

The following algorithm packages were used for analysis: SPIA [20], CEPA [21], GRAPHITE [22], PIANO [23], GAGE [24], ESEA [25]. The following databases were employed: REACTOME (http://reactome.org/), BIOCARTA (http://www.biocarta.com/), please note that the biocarta server is not available anymore. NCI (http://www.ndexbio.org/#/), KEGG (http://www.genome.jp/kegg/) [26, 27], MSigDB (http://software.broadinstitute.org/gsea/index.jsp) (H: hallmark gene sets, CP:BIOCARTA: BioCarta gene sets, CP:KEGG: KEGG gene sets, CP:REACTOME: Reactome gene sets). Unless otherwise specified pathway databases included in these packages were used. For SPIA analysis pathways were downloaded directly from KEGG. For GAGE and PIANO, annotation sets were downloaded from the Molecular Signatures Database v5.2 (http://software.broadinstitute.org/gsea/msigdb).

After SPIA analysis with the KEGG annotation database, pathways and biological processes most likely to be affected were selected after filtering results by criteria - pG < 0.05, NDE > 3. pG represents the combined *p*-value from gene enrichment and probability of perturbation accumulation in the pathway and NDE represents differentially expressed genes per pathways. The same criteria were applied for Graphite “runSpia” analysis with the Reactome, Biocarta and NCI annotation databases. For Piano gene set enrichment analysis, an adjusted *p*-value of < 0.05 for up and down regulated gene sets was set as filtering criterion. Minimum and maximum number of genes per set was defined as 3 and number of DE/5. Piano analysis was run using MSigDB “Hallmark gene sets” (h.all.v5) and “CP:BIOCARTA: BioCarta gene sets”, “CP:KEGG: KEGG gene sets”, “CP:REACTOME: Reactome gene sets” annotation sets. In case of CEPA pathway analysis, affected pathways were selected if 3 out of 6 (equal.weight, in.degree, out.degree, betweenness, in.reach, out.reach) statistics were *p*-value < 0.05 for all annotation databases used. For GAGE analysis log_2_-fold change for all genes after filtering were used and a *p*-value < 0.05 was set as filtering criterion for the results. For ESEA analysis, the expression matrix of all genes after filtering was used. NOM *p*-value was used as significant criterion for Gain-of-correlation and Loss-of-correlation result filtering. Affected pathways and biological processes that were not detected at least by 2 different methods with the same annotation database were filtered out. Only changes that occurred in both LIHC and KIRC patients with low ALDH7A1 expression were kept. KEGG pathway maps were rendered with “Pathview” [28].

### ^1^H NMR spectrometry

Twelve control and twelve ALDH7A1-depleted cell samples from 3 independently transduced polyclonal cell lines were analyzed in duplicate. Samples were extracted in chloroform-methanol-water [29]. The aqueous supernatant was lyophilized and stored at − 80 °C. Immediately before measurement, samples were rehydrated in 200 μl of 50 mM phosphate buffer (pH 7.4) in D_2_O, and 180 μl was transferred to a 3 mm NMR tube. The buffer contained the chemical shift reference 3-(trimethylsilyl)-propionic acid-D4, sodium salt and NaN_3_.

NMR measurements were performed at 25 °C on a Bruker Avance III HD 800 spectrometer, operating at a ^1^H frequency of 799.87 MHz, equipped with a 3 mm TCI cold probe. ^1^H NMR spectra were acquired using a single-90°-pulse experiment with a Carr-Purcell-Meiboom-Gill (CPMG) delay added, in order to attenuate broad signals from high-molecular-weight components. The total CPMG delay was 194 ms and the spin-echo delay was 4 ms. The water signal was suppressed by excitation sculpting. A total of 128 transients of 32 K data points spanning a spectral width of 20 ppm were collected, corresponding to a total experimental time of 6.5 min. The spectra were processed using iNMR (http://www.inmr.net). An exponential line-broadening of 0.5 Hz was applied to the free-induction decay prior to Fourier transformation. Spectra were referenced to the TSP signal at − 0.017 ppm, automatically phased and baseline corrected.

Drifting baseline of NMR spectra was corrected using the “rollingBall” algorithm. Spectra from BJ-4F3 cells were normalized against total intensity by dividing each intensity value by the sum of all intensity values. This method was chosen since total concentration of metabolites should be comparable across all samples. However, in our case spectra contained large peaks with significant variation between control and ALDH7A1 down-regulated cells, which could drastically affect total intensity values. Therefore, spectra were normalized against total intensity of a spectral region (above 4) that does not contain large peaks with significant variation. The “CluPA” algorithm was used to align peaks. Baseline correction, normalization and peak alignment was done using R package “ChemoSpec” (https://cran.r-project.org/web/packages/ChemoSpec/). Principal Component Analysis with “pareto” scaling was performed using R package “muma” [30]. One sample was excluded from analysis due to technical problems. Two samples were defined as outliers in the PCA analysis and were therefore also excluded.

To identify metabolites that are changed in ALDH7A1 depleted cells, the intensity values for signals above the baseline threshold defined as mean + 1SD of all intensity signals were compered. Non-parametric pairwise Wilcoxon-Mann Whitney U test with Benjamini-Hochberg correction for multiple testing was used to calculated *p*-values.

In the case of HUH7 and CAKI2 cells, ^1^H NMR spectra were processed and analyzed as above with minor adjustments. Six control and six ALDH7A1-depleted cell samples from 3 independently transduced cell lines were analyzed. Spectra were normalized against total intensity of a spectral region (above 1.5). “CluPA” algorithm was used to align peaks. “Rolling ball” algorithm (span – 50) was applied to correct shifting baseline. Baseline correction, data binning (bin = 4), normalization and peak alignment was done using R package “ChemoSpec”.

### Gene expression clustering

PPAR transcriptional targets were selected from KEGG database (http://www.genome.jp/kegg-bin/show_pathway?hsa03320). Low expressed genes were filtered out. Unsupervised hierarchical clustering analysis was applied to cluster LIHC and KIRC patient normal and tumor tissues into groups based on median centered log_2_ PPAR target gene expression values. Control and tumor samples were clustered separately.

### Immunohistochemistry

Liver and kidney cancer arrays presenting tumors and adjacent normal tissue biopsy samples were obtained from US Biomax (Rockville, MD, USA; HLiv-HCC180Sur-02, HLiv-HCC180Sur-03 and HKid-CRC180Sur-01). Additionally, 72 archival patient samples from the pathology department, Rigshospitalet Copenhagen were examined. Ethical approval was obtained from the Danish National Committee on Biomedical Research Ethics. Immunostaining was performed using rabbit anti-ALDH7A1 (Sigma: HPA023296) [31] and the streptavidin–biotin peroxidase complex method according to the manufacturer’s instructions (UltraVision HRP DAB system, Thermo). Sections were examined by an experienced pathologist to confirm the tissue identity and assigned a score: 0 (no staining), 1 (weak staining up to 10% of tissue), 2 (weak staining 10–25% of tissue), 3 (weak to moderate staining ≥50% of tissue), 4 (moderate to strong staining of 50–75% of tissue) and 5 (moderate to strong staining > 75% of tissue). The score for each tumor was calculated by subtracting the score of the normal tissue from that of that tumor.

#### Multivariate regression analysis

Patients with complete set of information on survival time, status, stage and ALDH7A1 regulation were included in the study. Hepatocellular carcinoma patients with stage I (7) and stage IV (3) disease were excluded from multivariate analysis due to small sample size. We also excluded patients with hepatic cirrhosis. All covariates were tested for the proportional hazards assumption, and the multivariate Cox proportional hazards regression models were created using R package “Survival” (https://cran.r-project.org/package=survival). Different models were compered by Likelihood ratio test and chi-square test. A forest plot was produced from the regression model with R package “forestmodel” (https://cran.r-project.org/web/packages/forestmodel). Likelihood ratio test were used to calculate *p*-values for the Kaplan Meier plots.

## Results

### ALDH7A1 depletion promotes invasive cell migration

**Fig. 1:**
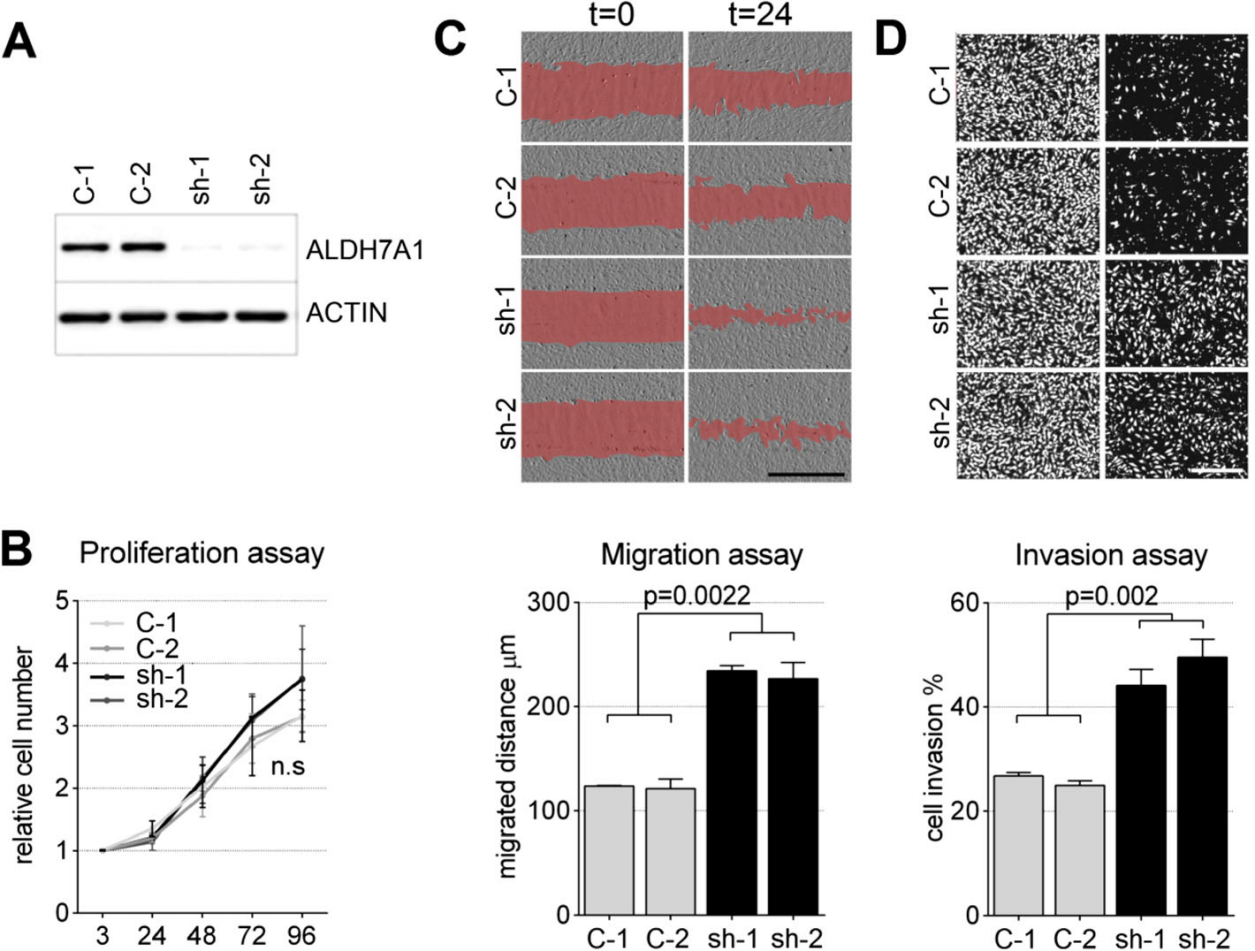
Behavior of ALDH7A1-depleted cells. **a** Immunoblot of BJ-4F3 cells transduced to express two independent shRNAs targeting ALDH7A1 mRNA (sh-1 and sh-2). Control 1 (C-1) indicates cells transduced with the empty vector. Control 2 (C-2) expressed a non-targeting shRNA. The blot was probed with anti-ALDH7A1. Anti-ACTIN was used as loading control. **b** Proliferation of BJ-4F3 cells treated as in (**a**). Cell number was measured by counting DAPI labeled nuclei. X-axis: time in hours, y-axis: relative cell number. Data represent average ± standard error of the mean (SEM) from 3 independent experiments normalized against number of plated cells. ns: the difference was not statistically significant. The two-tailed Mann Whitney test was used to calculate *p*-values. **c** Scratch assays to measure cell migration. Images show cells at *t* = 0 and 24 h. The empty area is shaded red for better visibility. Scale bar: 1000 μm. Average migration distance is shown in μm after 24 h for three independent experiments ± SEM in the panel below. The two-tailed Mann Whitney test was used to calculate *p*-values. **d** Matrigel transwell migration assay to measure cell invasiveness. Images show cells labeled with DAPI. Left panels show all cells on the top and bottom surface of the assay well after 24 h. Right panels show cells that successfully migrated through the matrix, which was removed for imaging. The percent of cells that crossed the gel barrier is shown below (average of 3 independent experiments ± SEM). The two-tailed Mann Whitney test was used to calculate *p*-values

Using an in vivo Drosophila tumor model, we identified an aldehyde dehydrogenase as a potential tumor suppressor that cooperated with EGFR (Additional file 1: Figure S1). ALDH7A1 is the presumptive human orthologue of the Drosophila enzyme (www.flybase.org). To investigate the role of ALDH7A1 in human cells, we first made use of partially transformed primary human BJ cells, which serve as a model for oncogene-dependent cellular transformation [32]. These cells have the advantage of a defined genetic background, free of the diverse mutational backgrounds and selection pressures associated with established cancer cell lines, but they show the increased migratory and invasive and anchorage-independent growth behaviors associated with cancer cells. BJ cells were stably transduced to express shRNAs targeting ALDH7A1. Depletion of ALDH7A1 was efficient using two independent shRNAs (Fig. 1a). This had little or no effect on cell proliferation (Fig. 1b), but ALDH7A1-depleted cells showed a significant increase in migration using an in vitro wound healing ‘scratch’ assay (Fig. 1c). ALDH7A1-depleted cells also showed an increase in invasiveness using a Matrigel trans-well invasion assay (Fig. 1d). These changes in cell behavior prompted us to examine ALDH7A1 levels in human cancer.

### ALDH7A1 mRNA levels correlate with clinical outcome in liver and kidney cancer

**Fig. 2:**
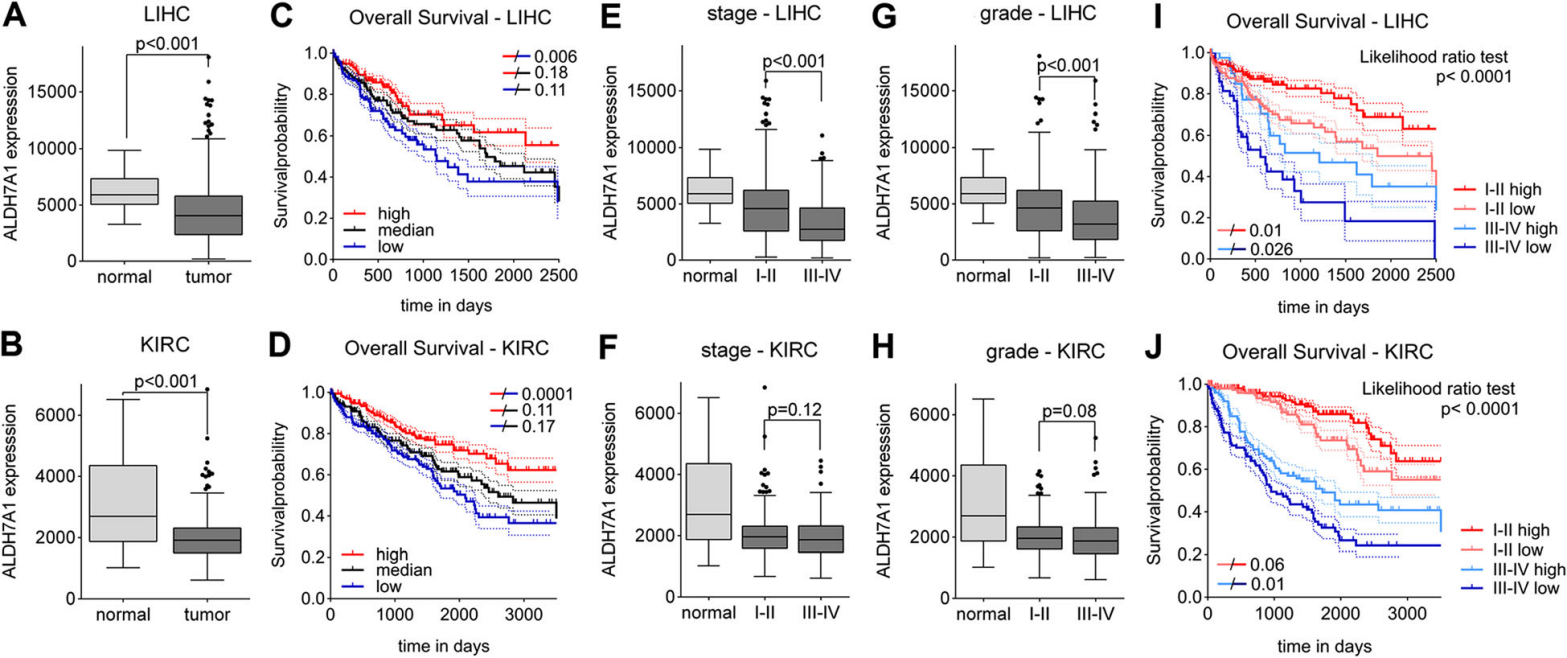
ALDH7A1 mRNA levels in liver (LIHC) and kidney (KIRC) cancer. **a**, **b** ALDH7A1 mRNA levels in normal (control) and tumor tissue from the LIHC and KIRC TCGA datasets. RSEM (RNA-seq expectation by maximization) reads are plotted on the Y-axis. *P*-values were calculated with the Mann–Whitney U test. **c**, **d** Kaplan-Meier plots showing overall survival of LIHC and KIRC patients as a function of ALDH7A1 mRNA expression. Patients were divided into three equal groups based on ALDH7A1 mRNA expression levels. Blue = lower 1/3; Black = middle 1/3; red = top 1/3. *P*-values were determined by Cox proportion hazard regression models. **e**-**h** ALDH7A1 mRNA levels as a function of tumor stage (**e**, **f**) and grade (**g, h**) for LIHC and KIRC patients. The two-tailed Mann Whitney test was used to calculate *p*-values. **i**, **j** Kaplan-Meier plots showing overall survival of LIHC and KIRC patients as a function of ALDH7A1 mRNA expression and stage. Patients were divided into early (stage I and II) and late (stage III and IV) stage groups. Each group was divided into two subgroups based on ALDH7A1 expression (at median). Significance was determined using the likelihood ratio test

Expression data was examined for 17 human cancer types from the TCGA database. ALDH7A1 mRNA was significantly lower in tumors from 7 cancers compared to normal tissue controls (Additional file 1: Figure S2). To ask if low ALDH7A1 mRNA expression correlated with clinical features, the patient population for each tumor type was subdivided into lower, middle and top thirds based on ALDH7A1 mRNA level. A significant reduction in overall survival was observed in the low expressing patient cohorts for hepatocellular carcinoma (LIHC, Fig. 2a, c) and for renal clear cell carcinoma (KIRC, Fig. 2b, d), but not for the other cancers (Additional file 1: Figure S2). ALDH7A1 levels were significantly lower in LIHC tumors of more advanced stage and histological grade (Fig. 2e, g). Within early and late stage groups, patients with low ALDH7A1 expression showed worse survival outcome: 5-year survival probability for the patients with late stage cancer and high ALDH7A1 expression was ~ 40% vs ~ 20% for patients with low ALDH7A1 (Fig. 2i). Similar results were obtained for KIRC, with low ALDH7A1 expression linked to poor survival for both early and advanced stage patients (Fig. 2j), although there was no significant difference in ALDH7A1 levels between the early and more advance stage or grade kidney cancer patients (Fig. 2f, h).

To ask how ALDH7A1 levels correlate with EGFR activity in these cancers, we used reverse-phase protein array data on EGFR phosphorylation, which provides a measure of EGFR pathway activation in the tumors. ALDH7A1 expression positively correlated with EGFR phosphorylation status in LIHC, but not KIRC. We performed Cox proportional hazards regression analysis to assess the association between ALDH7A1 mRNA and EGFR levels (Additional file 1: Figure S2). For LIHC, survival was significantly worse for patients with low ALDH7A1 in the high EGFR activity group, while it was not significant in low EGFR group. This was not the case for KIRC: low ALDH7A1 expression was significantly associated with poor clinical outcome in both low and high EGFR RNA expression groups. These findings suggest that the effect of low ALDH7A1 is closely linked with EGFR activity in liver cancer, but not in kidney cancer. Overall, these findings suggest that low ALDH7A1 expression might be a useful independent predictor of clinical outcome in liver and kidney cancers.

### Pathway and gene set enrichment analysis of LIHC and KIRC patients with low ALDH7A1

**Fig. 3:**
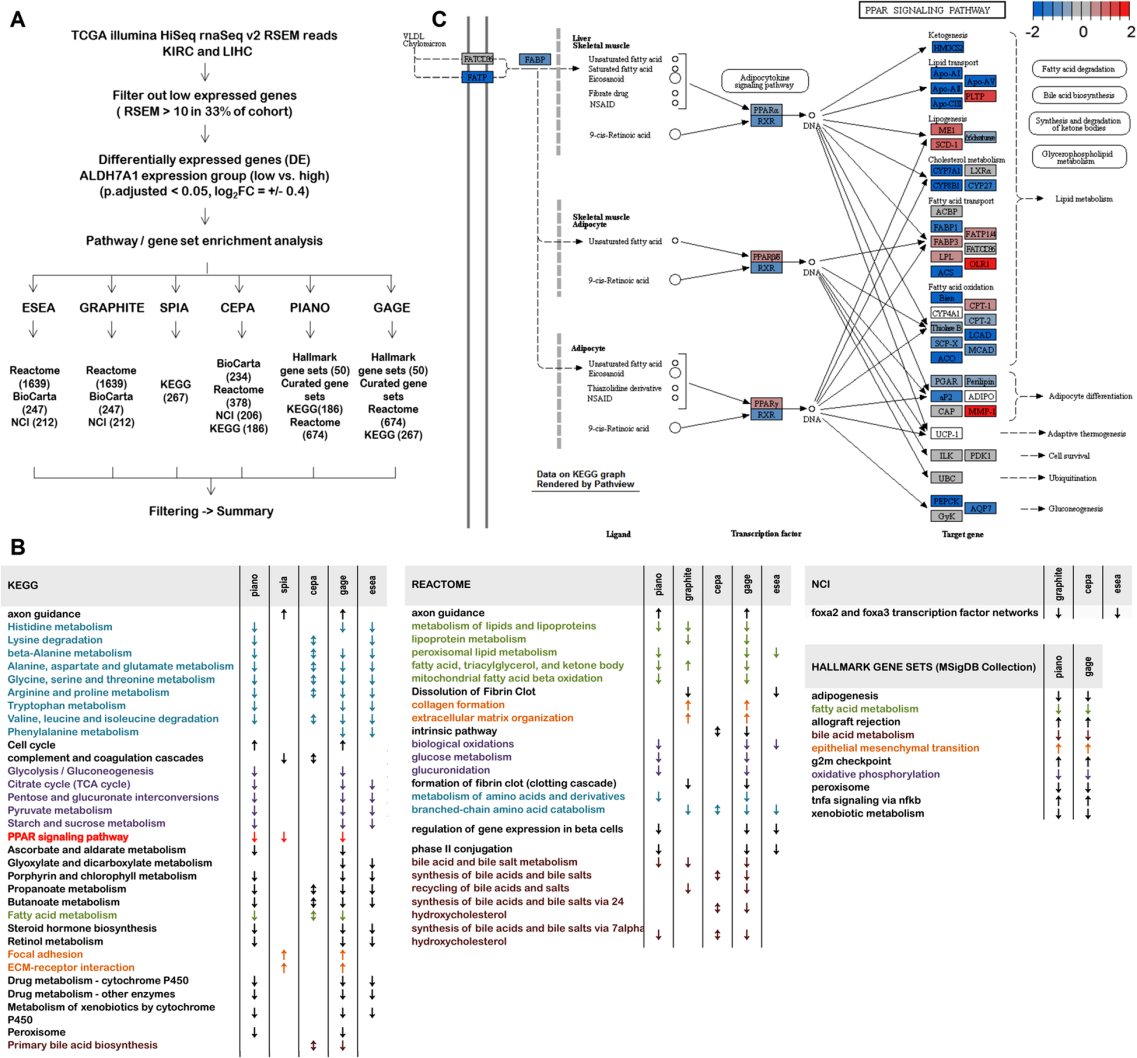
Pathway and gene set enrichment analysis. **a** Workflow of pathway and gene set enrichment analysis between ALDH7A1 high and low expression groups of LIHC and KIRC patients. Low expressed genes were filtered out by requiring RSEM > 10 in > 33% of patients. Genes differentially expressed between the low and high ALDH7A1 mRNA tumor groups were selected using a minimum threshold of +/− 0.4 log_2_-fold change, with an adjusted *p*-value of < 0.05. Fold change and *p*-values were used according to requirements of each specific method. Six different R packages (ESEA, GRAPHITE, SPIA, CEPA, PIANO and GAGE) were applied to the Reactome, BioCarta, NCI and KEGG pathway databases and the MsigDB gene set annotation collections, as indicated. **b** Summary of significantly affected pathways and biological processes after filtering. ↓ - pathway inactivated /down regulated; ↑ pathway activated /up regulated, ↕ - direction not provided. Light blue – amino acid metabolism; bright red – PPAR signaling pathway; light violet – energy metabolism; dark red – bile acid metabolism; light green – lipid, fatty acid metabolism; light orange - processes related to cell motility. **c** Map of the PPAR signaling pathway from the KEGG database, showing genes that were differentially expressed in LIHC tumors with low ALDH7A1 expression. Scale – log_2_FC; Blue: lower expression in tumors. Red: higher expression in tumors. Image rendered by Pathview [28]

To generate hypotheses about possible causes of the poor outcome in the low-ALDH7A1 LIHC and KIRC patient groups, we analyzed TCGA gene expression data and performed pathway analysis to compare tumors from the patient groups with top versus bottom third ALDH7A1 expression. Multiple reference databases and analytical tools were applied, since no accepted standard exists for this field at this time [33, 34]. These are summarized in Fig. 3(a). After filtering, we focused on changes that were found in both the LIHC and KIRC tumor comparisons and that were captured by at least two algorithms (Fig. 3b, Additional file 1: Figure S3A). Pathways and gene sets associated with extracellular matrix, cell adhesion and epithelial-mesenchymal transition were upregulated in the low-ALDH7A1 tumors (Fig. 3b). This was intriguing in light of the changes in cell migration and invasion that resulted from depletion of ALDH7A1. Many pathways and gene sets involved in cellular metabolism were downregulated in the low-ALDH7A1 tumors. These included energy metabolism, amino acid metabolism, lipid and fatty acid metabolism and bile salt metabolism. This analysis also showed a significant correlation between low ALDH7A1 expression and lower PPAR signaling activity. This was noteworthy because PPARs are transcription factors that regulate cellular metabolism. Figure 3(c) illustrates changes in the expression levels of genes regulated by PPAR isoforms in the LIHC data set. The corresponding data for KIRC and data for other pathways are provided in Additional file 1: Figure S3B. If this relationship is causal, ALDH7A1 activity levels could act via PPARs to affect a number of metabolic pathways and cellular phenotypes.

### Metabolic profiling

**Fig. 4:**
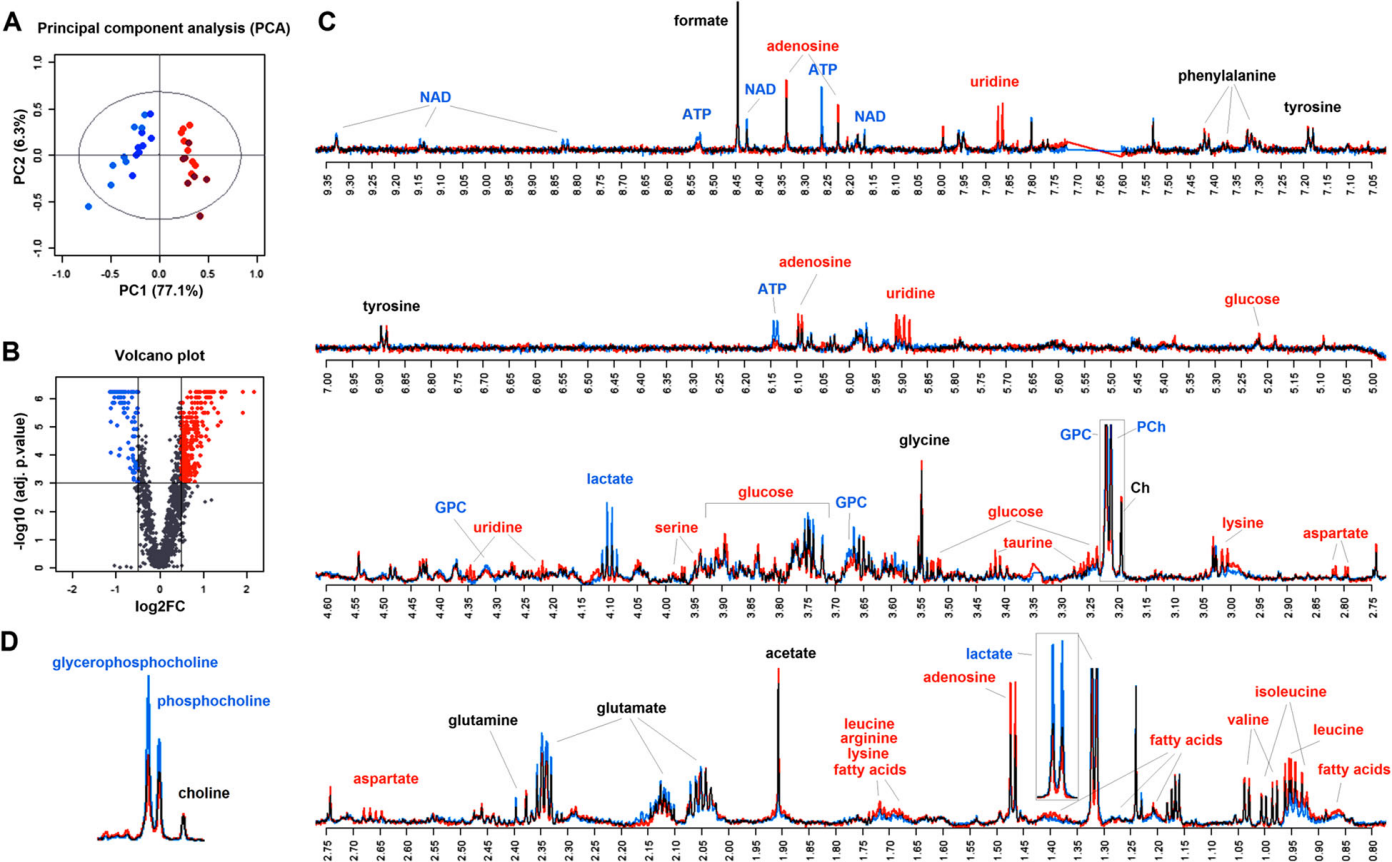
Metabolite profile of control and ALDH7A1-depleted primary cells. **a** Principal component analysis score plot with pareto scaling. First and second component scores were plotted. Blue dots show control cells (C1 and C2). Red dots show ALDH7A1-depleted cells (sh1 and sh2). The first PCA component accounted for 77% of the variation and significantly discriminated between control and ALDH7A1-depleted cells. **b** Volcano plot of significance versus log_2_-fold change of all intensity points of the spectra above background. The x axis shows log_2_FC between control and ALDH7A1-depleted cells of all intensity points, while the y axis shows the odds ratio (−log_10_(adjusted *p*-value)). Significantly increased and decreased spectral points are shown in red and blue, respectively. Adjusted *p*-value threshold < 0.001, log_2_FC +/− 0.5. **c** ^1^H NMR spectra. Average spectra of all control (blue) and ALDH7A1-depleted cell samples (red). All assigned metabolite signals are indicated. Significantly upregulated metabolites appear as red. Significantly down-regulated metabolites appear as blue. Overlap, indicating no change, appears black. **d** Detail showing region (3.15–3.25) containing the identified phosphocholine and glycerophosphocholine signals

As an independent approach to explore the impact of ALDH7A1-depletion, metabolic profiling was performed on aqueous extracts from control and ALDH7A1-depleted primary BJ cells using high-resolution ^1^H NMR spectroscopy. Metabolite spectra were normalized, peaks aligned and unsupervised principal component analysis was performed to assess variation between the control and ALDH7A1-depleted cells. This analysis showed a clear separation between control cells and the cells depleted of ALDH7A1, indicating major differences in metabolite composition (Fig. 4a). Figure 4(b) shows a volcano plot of log_2_-fold change vs. odds ratio (−log_10_(adj.p.value)) for all spectral points above background, to visualize the distribution and magnitude of significant changes in metabolite composition.

Twenty-six endogenous metabolites were identified by comparing chemical shifts to reference libraries [35, 36]. Eighteen of these were significantly different between control and ALDH7A1-depleted cells, with adjusted *p*-value < 0.001 and log_2_-fold changes +/− 0.5 (Fig. 4c). ALDH7A1-depletion led to increased levels of several amino acids, fatty acids and glucose, while NAD, ATP and lactate levels were lower (Fig. 4c). These changes are consistent with the pathway analysis in Fig. 3, indicating reduced degradation of branched chain amino acids and lysine, as well as changes in energy metabolism. Some of these pathways are regulated by PPAR signaling [15]. We also observed a decrease in glycerolphosphocholine (GPC) and phosphocholine (PC) levels in the ALDH7A1-depleted cells (Fig. 4d). Isoforms of GPC have been identified as activating ligands for PPARα [13, 14]. Metabolite analysis was also carried out on the liver carcinoma cell line HUH7 and on CAKI2 kidney cancer cells (Additional file 1: Figure S4). As in the primary cells, lactate levels decreased and glucose levels increased after ALDH7A1-depletion in both cell lines. GPC, PC and choline levels were also reduced in HUH7 cells (PC and GPC were not at detectable levels in the CAKI2 cells). Together with the data in Fig. 3, these observations suggested a link between PPAR activity and ALDH7A1-depletion.

### PPAR activity in LIHC and KIRC

**Fig. 5:**
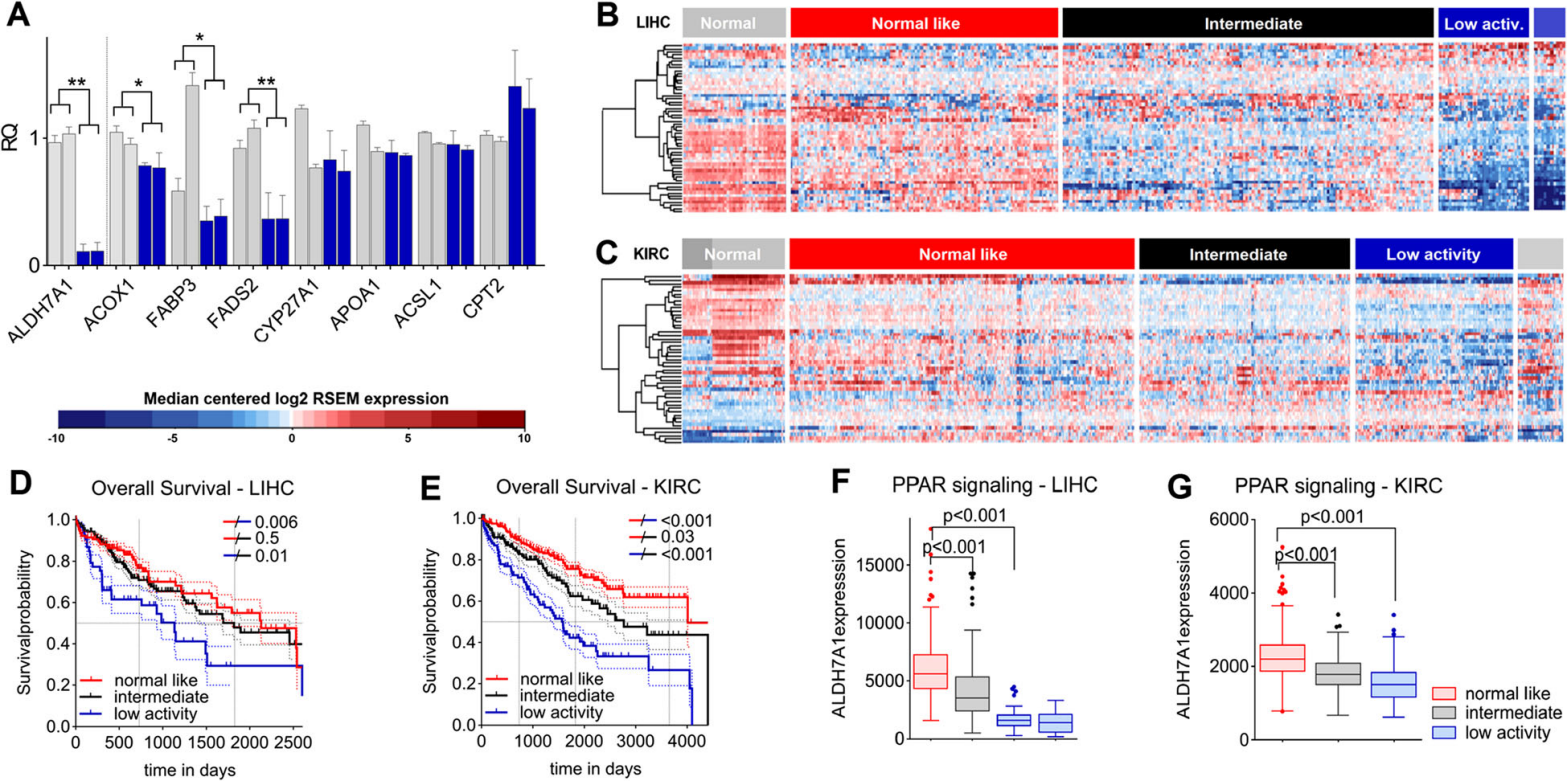
Correlation between PPAR signaling, ALDH7A1 levels and patient survival in LIHC and KIRC. **a** qPCR of PPAR transcriptional targets. Light grey – control BJ cells transduced with the empty vector and non-targeting shRNA, accordingly. Blue – ALDH7A1-depleted cells transduced with two independent shRNAs (sh-1 and sh-2). Data represent average ± standard error of the mean (SEM) from 3 independent experiments normalized β-ACTIN, KIF and TBP. The two-tailed Mann Whitney test with adjustment for False Discovery Rate was used to calculate *p*-values. **b**-**c** Heatmaps of median centered log_2_ RSEM expression values of PPAR target genes (selected from KEGG, version 6/3/16) for LIHC (**b**) and KIRC (**c**) data sets. Hierarchical clustering (Wards.D) was applied. X-axis patients, y-axis genes. Red – higher, blue – lower then median expression. **d**-**e** Overall survival analysis for patients divided into three groups based on the clustering analysis: normal-like (red), intermediate (black) and low PPAR activity (blue). Kaplan-Meier survival curves were plotted and Cox proportion hazards regression models were used to calculate *p*-values between groups. **f**-**g** Expression of ALDH7A1 mRNA levels in the patient groups defined as normal-like (red), intermediate (black) and low PPAR activity (blue). Boxplots indicate the mean RSEM value, upper and lower quartile. The two-tailed Mann Whitney test was used to calculate *p*-values

To test the hypothesis that ALDH7A1-depletion might act via PPAR activity, we examined the expression of PPAR targets in ALDH7A1-depleted cells. Figure 5(a) shows that the expression of several known PPAR target genes was reduced following ALDH7A1-depletion in primary BJ cells (Fig. 5a). Next we examined PPAR transcriptional activity in the LIHC and KIRC TCGA datasets. If the effects of low ALDH7A1 activity are mediated though regulation of PPAR activity, we should find a corresponding correlation between PPAR transcriptional activity and survival outcome in patient datasets. Tumors and control samples were clustered into groups based on expression of annotated PPAR targets (KEGG map version 6/3/16). PPAR target expression varied significantly among liver and kidney cancer patients. One group showed expression signatures resembling control samples for both tumor types (Fig. 5b, c, ‘normal-like’). A second group showed a ‘low activity’ signature, consisting of reduced expression of the PPAR targets that were high in the controls and reciprocally increases in some low-expressed targets (PPARs can regulate transcriptional activity positively as well as negatively). A third group showed intermediate expression levels. Patients with the low PPAR activity profile had lower survival in both tumor types (Fig. 5d, e). For LIHC patients the survival difference between the normal-like and low PPAR groups was significant (*p* = 0.006). For KIRC patients, the range of survival outcomes was larger, and all pairwise combinations were significantly different. These results indicate that PPAR signaling activity can predict poor clinical outcome, suggesting the importance of this pathway in aggressive liver and kidney cancers.

Interestingly, the low PPAR activity group also showed significantly lower expression ALDH7A1 (Fig. 5f, g). This observation supports the hypothesis that the metabolic consequences of low ALDH7A1 activity are a significant cause of the low PPAR activity in these tumors.

To further investigate this correlation, we examined the 5 cancer types with lower ALDH7A1 expression, that did not show worse survival outcome (Additional file 1: Figure S5). None of the 5 showed a correlation between low PPAR activity and low ALDH7A1 expression, and there was no decrease in overall survival probability compared to the “normal-like” PPAR group for 4 of them. Thus, low ALDH7A1 expression does not seem to be causally linked to low PPAR activity in other cancers. Nor does low PPAR activity always correlate with low survival in other cancers. The three-way correlation between ALDH7A1 expression, PPAR activity and clinical outcome appears to be a feature of kidney and liver cancers, but not other cancer types.

### Activation of PPARs rescues ALDH7A1-depleted cell phenotypes

**Fig. 6:**
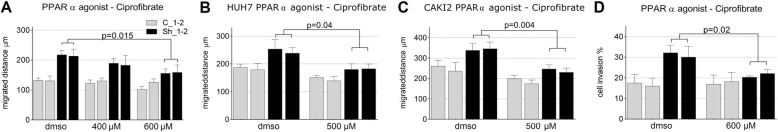
Effect of PPAR agonists on the cell invasion and migration phenotype of ALDH7A1-depleted cells. **a**-**c** Quantification of wound healing assays after 24 h migration. **a** Primary BJ cells, HUH7 and **c** CAKI2 cancer cells were treated with PPARα agonist or DMSO as a control. The migrated distance was measured (μm), and averages from three independently transduced cell lines were calculated (± SEM). **d** Quantification of cell invasion of primary BJ cells through Matrigel over 24 h. The bar plots show the percent of cells that crossed the gel barrier (average of 3 independent experiments ± SEM). The two-tailed Mann Whitney test was used to calculate *p*-values

If the invasive and migration phenotypes observed in ALDH7A1-depleted cells are due to reduced activation of PPARs, we reasoned that restoring PPAR activity by treating cells with activating ligands should result in more normal cell behavior. The PPARα agonist Ciprofibrate lowered the migration of ALDH7A1-depleted BJ cells while having little effect on the migration of control cells in the scratch assay (Fig. 6a). Treatment with the PPARα agonist Ciprofibrate did not affect the level of ALDH7A1 protein in these cells, but Ciprofibrate treatment was effective in restoring PPAR target gene expression in ALDH7A1-depleted cells to a level comparable to the control cells (Additional file 1: Figure S6). The PPARβ agonist GW501516 also restored migration to near normal levels, while the PPARγ agonist Rosiglitazone did not (Additional file 1: Figure S6). To extend these results, we tested ALDH7A1-depletion in several cancer cell lines (Additional file 1: Figure S7). Among those, HUH7 and CAKI2 showed reduced expression of PPAR targets after ALDH7A1-depletion. The PPARα agonist also normalized the migration of the ALDH7A1-depleted HUH7 and CAKI2 cells in the scratch assay (Fig. 6b, c). BJ cells treated with the PPARα agonist showed a decrease in transwell invasive migration, toward that seen in the control cells (Fig. 6d); the PPARβ agonist GW501516 did not (Additional file 1: Figure S6). These data provide evidence that the behavioral changes that result from ALDH7A1-depletion can be offset by restoring PPAR activity, with PPARα agonists improving both invasive and scratch assay behaviors.

### ALDH7A1 protein levels predict clinical outcome

**Fig. 7:**
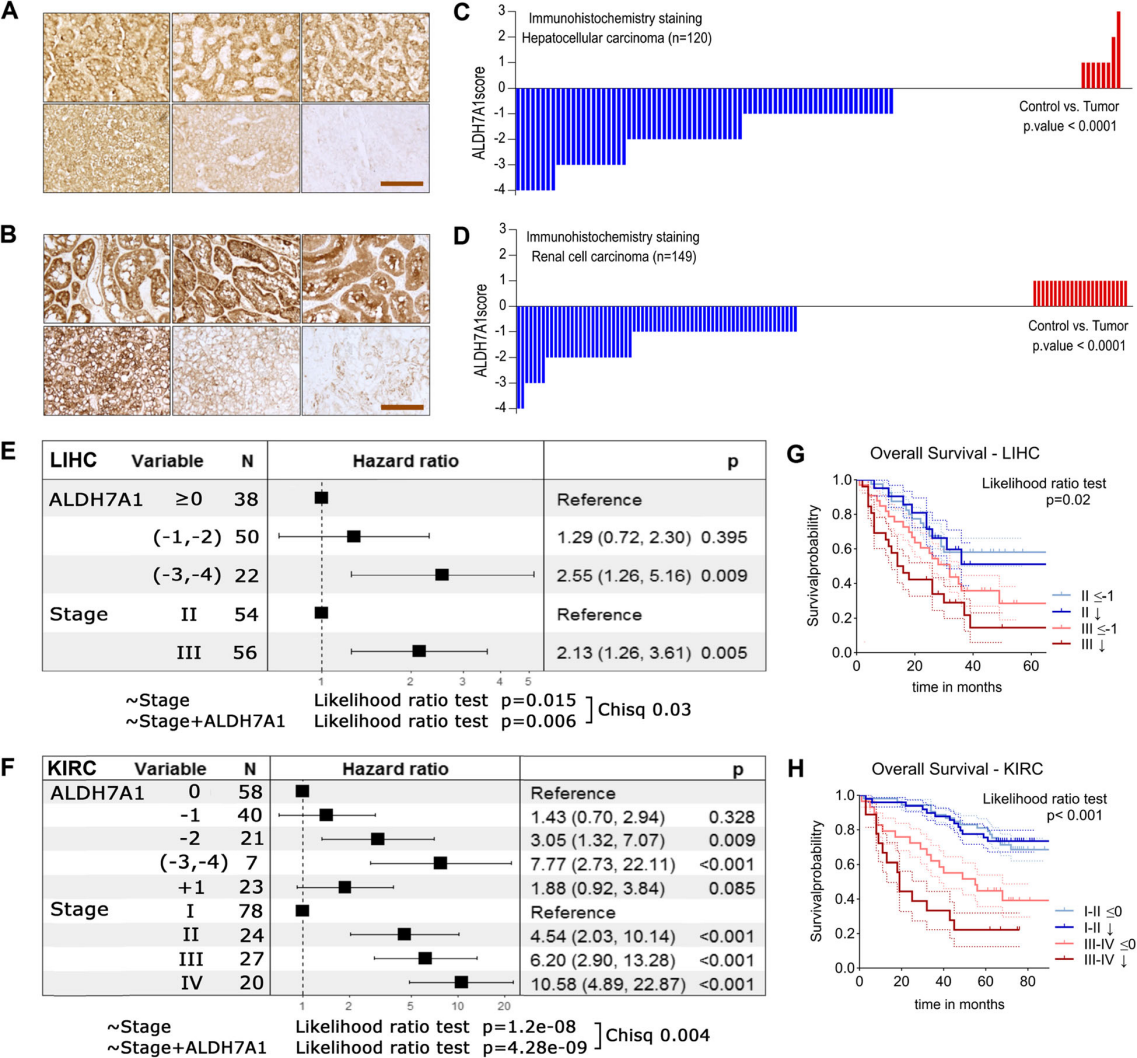
Association between ALDH7A1 protein levels and patient survival in liver and kidney cancer. **a**-**b** Representative images of hepatocellular carcinoma (HCC) and renal clear cell carcinoma (ccRCC) patient biopsies stained with anti-ALDH7A1. Scale bar: 100 μm. Upper panel adjacent normal tissue and lower panel corresponding tumor tissue from the same patient. **c**-**d** 120 hepatocellular carcinoma and 149 renal clear cell carcinoma tissue and paired adjacent normal tissue were labeled with anti-ALDH7A1 and scored for antigen expression. The staining score shown was calculated by subtracting the normal tissue score from the corresponding tumor score. The *p*-value was determined by the Wilcoxon matched-pairs signed rank test. **e**-**f** Forest plot representing multivariate Cox proportional hazards regression models of the hepatocellular carcinoma and renal clear cell carcinoma data scored in (**c-d**). For each variable the square and horizontal lines represent the estimated Hazard Ratio (HR) and corresponding confidence interval, respectively. The value of the model was estimated by the likelihood ratio test, and different models were compared by chi-square test. **g**, **h** Kaplan-Meier plots to visualize the association shown in (**e**) and (**f**). Survival plots show early and late stage patient groups divided by the ALDH7A1 immunohistochemistry score. The *p*-values were determined by likelihood ratio testing

The data provided thus far have shown that expression of ALDH7A1 and the PPAR target signature profile both correlate with patient outcome, and so could provide clinically relevant information. However, expression profiling is not in routine use as a clinical diagnostic tool, so an approach based on immunohistochemistry (IHC) would have advantages. We used ALDH7A1 antibody IHC on tissue arrays that pair the tumor samples with adjacent normal tissue for hepatocellular carcinoma (HCC) and renal clear cell carcinoma (ccRCC). For kidney cancer we also included 72 archival patient samples. Representative images are shown in Fig. 7(a, b). For HCC, we observed lower ALDH7A1 levels in 62% of tumors compared to the adjacent normal tissue (Fig. 7c: IHC score for tumor minus the score for normal tissue). For ccRCC, the ALHD7A1 score was low in 46% of samples (Fig. 7d). Information about the patients is compiled in Additional file 1: Figure S8.

Using these data, we asked whether the ALDH7A1 IHC score (tumor-normal) was an informative parameter in the context of well established clinical parameter such as stage for patient survival using multivariate Cox proportional hazards regression analysis. Regression analysis showed a significantly increased hazard ratio for the patients with low ALDH7A1 scores and for those with higher stage for both cancers (Fig. 7e, f). Next, we compared two regression models, one based solely on tumor stage, and one that incorporated the ALDH7A1 score in addition to tumor stage. The likelihood ratio test showed that including the ALDH7A1 parameter added significant value to the model’s predictive power (chi-square test: *p* = 0.03 and *p* = 0.004; Fig. 7e, f).

Kaplan-Meyer curves were used to visualize patient survival as a function of stage and ALDH7A1 IHC score (Fig. 7g, h). Within the lower-stage groups, there was no significant difference in survival with ALDH7A1 score. However, in the patients with more advanced stage tumors, a low ALDH7A1 score correlated with significantly reduced survival probability. Differences between groups were statistically significant (likelihood ratio test *p* = 0.02 for HCC and *p* < 0.001 for ccRCC). On this basis, we propose that IHC scoring for ALDH7A1 protein, combined with standard histopathological criteria may provide prognostic information on survival probability to identify HCC and ccRCC patients with more aggressive disease.

## Conclusions

To date, little is known about the role of ALDH7A1 in cancer. Metabolic roles of ALDH7A1 include protecting cells from oxidative stress by metabolizing aldehydes derived from lipid peroxidation [37], and protecting cells from osmotic stress by metabolizing betaine aldehyde to betaine, which serves as a cellular osmolyte [38]. Our metabolic profiling data now links ALDH7A1 activity to the levels of activating ligands for the PPAR transcription factors. We have provided evidence that the effects of ALDH7A1 on cellular migration and on invasive behaviors is mediated through decreased PPAR activity. These data suggest a mechanism by which the ALDH7A1 activity can influence a wide range of metabolic pathways and cellular functions, with the potential to impact disease progression.

Literature on the role of ALDH7A1 in cancer suggests a somewhat complex scenario, with different outcomes in cancers of different tissue origin. In some reports, high ALDH7A1 has been linked to more severe disease. Positive ALDH7A1 protein staining correlates with increased cancer recurrence in non-small cell lung carcinoma [39]. High ALDH7A1 protein expression has been reported in ovarian cancer, with highest expression in invasive ovarian cancer cells comparing to healthy ovarian epithelia [40]. On the other hand, our analysis of 17 cancer types, using TCGA RNAseq data showed that ALDH7A1 expression was lower in several cancer types and that lower expression correlated with poor clinical outcome for HCC and renal ccRCC. ALDH7A1 activity impacts a number of metabolite pathways directly, and acts indirectly via PPARs on others. These metabolic shifts appear to impact different types of cancer differently. Why are liver and kidney cancer sensitive to the effects of low ALDH7A1? ALDH enzyme family members have distinct activities and substrate specificities. ALDH7A1 expression is high in the metabolically active kidney and liver tissues, whereas lung and prostate tissue express only low or moderate ALDH7A1 levels. ALDH7A1 is also to protect cells from osmotic stress. This might be important in liver and kidney, where turnover of osmolites such as betaine and glycerophosphocholine are tightly regulated. If high ALDH7A1 expression is important for liver and kidney homeostasis, it is tempting to speculate that low expression of this enzyme might contribute to cancer development in these tissues to a greater extent than in other tissue types.

### PPARs as therapeutic targets for cancer

PPARs are ligand activated transcription factors that play an important role as regulators of metabolism and cellular homeostasis. PPARs are known to regulate fatty acid synthesis, uptake and storage, mitochondrial and peroxisomal fatty acid oxidation and ketogenesis, insulin sensitivity, glucose metabolism, drug metabolism and amino acid metabolism. In addition, PPARs have anti-inflammatory and immune suppressive functions. Given these wide-ranging effects on cellular metabolism and defense mechanisms, it may not be surprising that PPARs have been implicated as oncogenes in some cancer models and as tumor suppressors in others [41, 42].

PPAR agonists are in clinical use for metabolic disorders and have been considered as cancer therapeutics. However, a number of safety concerns have been raised due to unwanted side effects and cancer development in rodent models [17]. PPAR activators used as dietary supplements induced liver enlargement accompanied by oxidative stress in rats and mice [43]. The PPARγ agonist Rosiglitazone was withdrawn due to increased risk of myocardial infarction [44]. A meta-analysis found a modest but clinically significant increase in overall risk of bladder cancer upon long term treatment of another PPARγ agonist Pioglitazone [45]. However, two large meta-analysis studies showed no statistically significant association between Fibrate (PPARα agonist) and cancer incidence [46, 47]. To exploit the potential of PPARs as drug targets for cancer, we will require a more nuanced understanding of the role of specific PPAR isoforms in specific cancers, as well as means to identify patient groups who might benefit from therapeutics targeting PPARs.

Our studies reveal a striking three-way connection between low ALDH7A1 abundance, low PPAR activity and poor clinical outcome. Notably, patients with low PPAR activity also have low ALDH7A1 levels, suggesting a causal link between these two. This is likely due to the effects of ALDH7A1 on PPAR ligand levels, and is reflected experimentally by reduced PPAR target expression in cells depleted of ALDH7A1. Low PPAR activity is a useful predictor of poor clinical outcome, but it is difficult to measure in a clinical setting. We have provided evidence that scoring for ALDH7A1 levels by IHC may be a useful surrogate for PPAR activity to predict clinical outcome for patients with HCC and ccRCC.

Clinical trials are evaluating PPARα activation for treating non-alcoholic fatty liver disease and primary biliary cirrhosis, in combination with existing treatments (ClinicalTrials.gov identifier: NCT00262964, NCT00575042, NCT02823353, NCT02965911, NCT02823366). A trial evaluating the effect of the PPARα agonist Fenofibrate on patients with multiple myeloma (NCT01965834) is ongoing. However, to our knowledge, trials evaluating the effects of PPARα agonists on patients with HCC or ccRCC have not been started. We propose that selecting HCC or ccRCC patients according to ALDH7A1 IHC status might be a promising avenue for future study.

## Additional file

Additional file 1: **Figure S1.** ALDH7A1 depletion promotes tumor formation in vivo**.** Describes effect of depleting Drosophila ALDH on tumor formation in vivo. **Figure S2.** ALDH7A1 mRNA level in human cancers. (A) Compares ALDH7A1 expression levels in TCGA datasets for 19 human cancers and compares survival outcome in low middle and high expressing patient groups. (B) Heatmap of the correlation between ALDH7A1 mRNA expression and EGFR RNA and EGFR phosphorylation in all cancer types. (C) Cox proportional hazard regression analysis of the association between ALDH7A1 mRNA and EGFR levels for liver and kidney cancer. **Figure S3.** Pathway analysis. (A) Gene set and pathway analysis comparing low vs high ALDH7A1 tumors. (B) Effects of low ALDH7A1 on pathways in KIRC. **Figure S4.** Metabolite profiles on cancer cell lines. **Figure S5.** Assessment of correlation between PPAR activity and ALDH7A1 on other cancers. Shows shows survival outcome for patients groups by PPAR target signature groups, and comparison with ALDH7A1 expression. **Figure S6.** Effects of PPAR agonists. Shows the effects of PPAR agonist treatment on ALDH7A1 protein levels, would healing assays, invasive migration (transwell) assays and PPAR target gene expression levels. **Figure S7.** Assays on cancer cell lines. Summarizes assays carried out on cancer cell lines. **Figure S8.** Clinical characteristics of the patients included in the study Summarizes TCGA clinical data. (PDF 40809 kb)

## Abbreviations

ALDH: Aldehyde dehydrogenase
ATP: Adenosine triphosphate
ccRCC: Kidney renal clear cell carcinoma
D_2_O: Deuterium oxide
DAB: Diamino benzidine
DAPI: 4′,6-diamidino-2-phenylindole
DMEM: Dulbecco’s modified eagle medium
DMSO: Dimethylsulfoxide
EGFR: Epidermal growth factor receptor
FCS: Fetal calf serum
GPC: Glycerolphosphocholine
HCC: Hepatocellular carcinoma
HRP: Horse radish peroxidase
hTert: Human telomerase
IHC: Immunohistochemistry
KEGG: Kyoto encyclopedia of genes and genomes
KIRC: Kidney renal clear cell carcinoma
LIHC: Liver hepatocellular carcinoma
mHz: MegaHertz
mRNA: Messenger RNA
NAD: Nicotinamide adenine dinucleotide
NAD(P): Nicotinamide adenine dinucleotide phosphate
NaN_3_: Sodium azide
NMR: Nuclear magnetic resonance
PC: Phosphocholine
PPAR: Peroxisome proliferator-activated receptor
qPCR: Quantitative real-time polymerase chain reaction
RNA: Ribonuceleic acid
RNAseq: RNA sequencing
RT-PCR: Quantitative real-time polymerase chain reaction
shRNA: Short hairpin RNA
TCGA: The cancer genome atlas
