# Supplementary material for: Metabolic control of PPAR activity by aldehyde dehydrogenase regulates invasive cell behavior and predicts survival in hepatocellular and renal clear cell carcinoma
Source: BMC Cancer. 2018 Nov 28;18:1180. doi: 10.1186/s12885-018-5061-7 (PMC6264057; doi:10.1186/s12885-018-5061-7)
Supplement: Supplementary file 1 — Figure S1. ALDH7A1 depletion promotes tumor formation in vivo. Describes effect of depleting Drosophila ALDH on tumor formation in vivo. Figure S2. ALDH7A1 mRNA level in human cancers. (A) Compares ALDH7A1 expression levels in TCGA datasets for 19 human cancers and compares survival outcome in low middle and high expressing patient groups. (B) Heatmap of the correlation between ALDH7A1 mRNA expression and EGFR RNA and EGFR phosphorylation in all cancer types. (C) Cox proportional hazard regression analysis of the association between ALDH7A1 mRNA and EGFR levels for liver and kidney cancer. Figure S3. Pathway analysis. (A) Gene set and pathway analysis comparing low vs high ALDH7A1 tumors. (B) Effects of low ALDH7A1 on pathways in KIRC. Figure S4. Metabolite profiles on cancer cell lines. Figure S5. Assessment of correlation between PPAR activity and ALDH7A1 on other cancers. Shows shows survival outcome for patients groups by PPAR target signature groups, and comparison with ALDH7A1 expression. Figure S6. Effects of PPAR agonists. Shows the effects of PPAR agonist treatment on ALDH7A1 protein levels, would healing assays, invasive migration (transwell) assays and PPAR target gene expression levels. Figure S7. Assays on cancer cell lines. Summarizes assays carried out on cancer cell lines. Figure S8. Clinical characteristics of the patients included in the study Summarizes TCGA clinical data. (PDF 40809 kb) [file 12885_2018_5061_MOESM1_ESM.pdf]

Additional file Figures – Andrejeva et al.

Figure S1: ALDH7A1 depletion promotes tumor formation in vivo

EGFR + GFP				control + GFP			
control		CG9629 RNAi		control		CG9629 RNAi	
# tested	# with tumors	# tested	# with tumors	# tested	# with tumors	# tested	# with tumors
57	0	59	45	60	0	45	0

Experimental design: To generate adult-specific, and spatially restricted transgene expression, UAS-transgenes were placed under apterous-Gal4 control. Gal4 activity was inhibited during larval and pupal development using the temperature sensitive form of $Gal80^{ts}$, by rearing animals at the permissive temperature (18°C). Newly emerged adult flies were shifted to 29°C, inactivating the Gal80 inhibitor and allowing the apterous-Gal4 transgene to direct expression of UAS-EGFR, together with a UAS-GFP marker to label the tissue. A UAS-RNAi transgene targeting CG9629 was used to test tumor formation in the context of EGFR overexpression (left half). GFP-expressing tumors were found in 76% of animals expressing EGFR and the CG9629 RNAi transgene. None were found in flies expressing EGFR alone or the CG9629 RNAi transgene alone.

This assay system has been used to identify context-dependent tumor suppressors based on transgene expression during larval stages (Herranz et al., 2012 Genes Dev 26, 1602-1611). Modification for use to screen for tumor formation in the adult will be described elsewhere (Kugler et al., in preparation)

Figure S2: ALDH7A1 mRNA level in human cancers

A

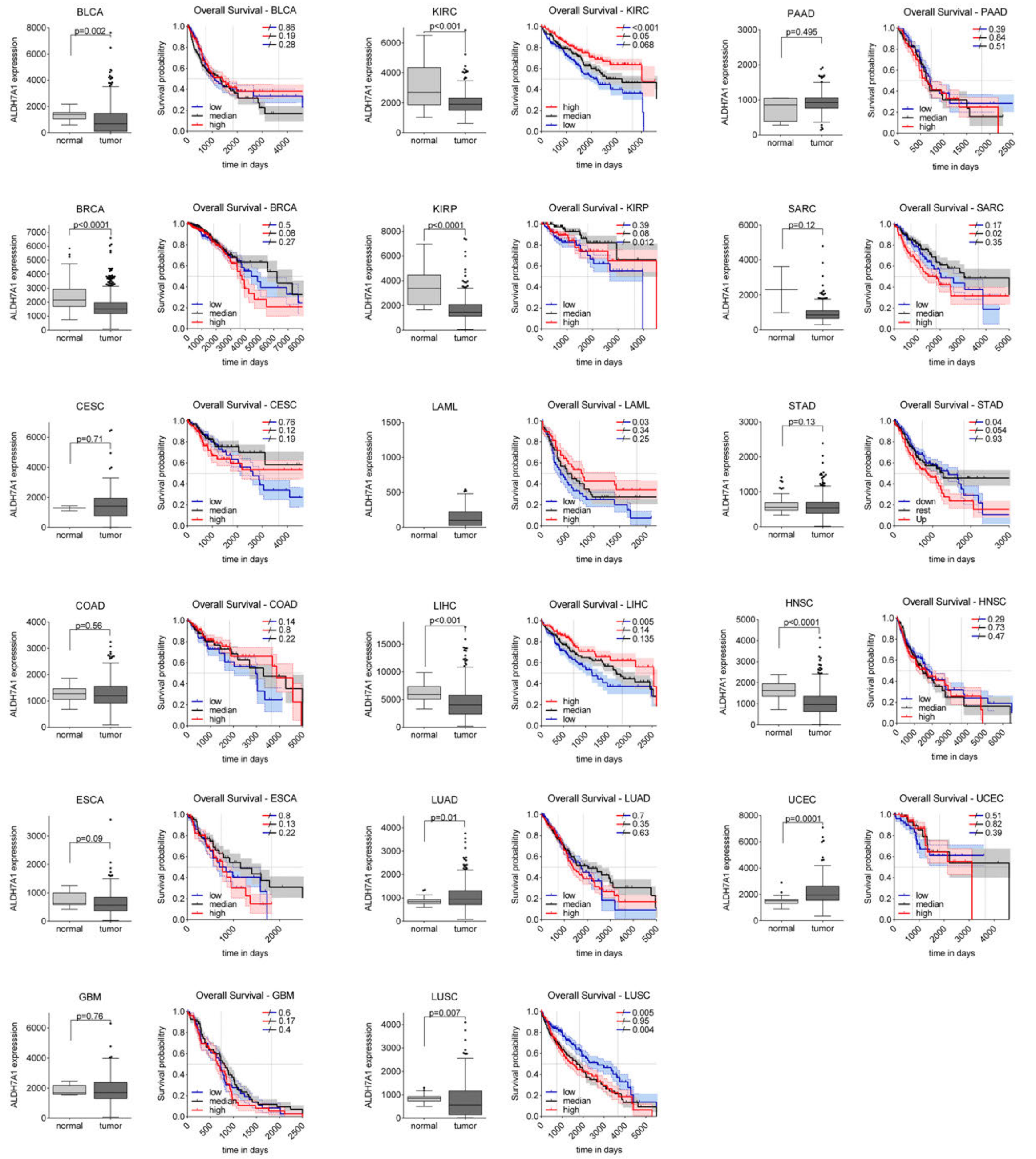

For each cancer type the box plot at left shows mRNA level in normal and tumor tissue, with mean RSEM (RNA-seq Expectation by maximization) and upper and lower quartile. Outliers were excluded for visualization. Two-tailed Mann Whitney test was used to calculate p-values. Right panels show Kaplan-Meier survival curves for patients with lower (blue), middle (black) and upper (red) thirds of ALDH7A1 mRNA expression. Cox proportion hazards regression models were used to calculate p-values between groups. Abbreviations are TCGA designations for cancer types.

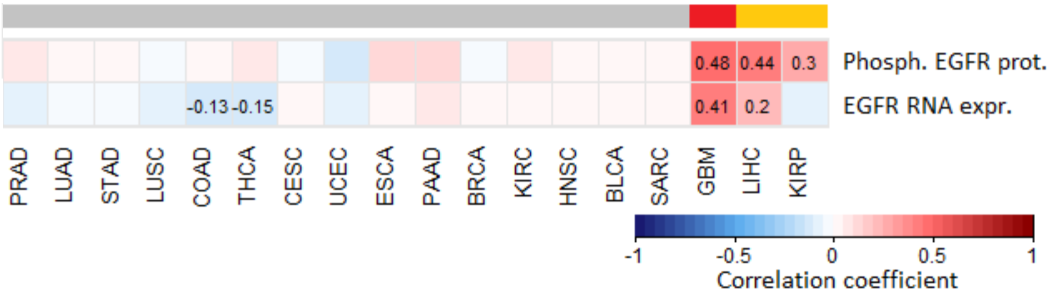

(B) Heatmap of the correlation between ALDH7A1 mRNA expression and EGFR RNA and EGFR phosphorylation in all cancer types. Red: positive correlation coefficients, blue: negative correlation. Correlations with significant p-values are indicated. ALDH7A1 expression positively correlates with EGFR phosphorylation status in GBM, LIHC and kidney (KIRP, but not KIRC), and with EGFR mRNA levels in GBM and LIHC. There is weak negative correlation in colon (COAD) and thyroid (THCA) cancer.

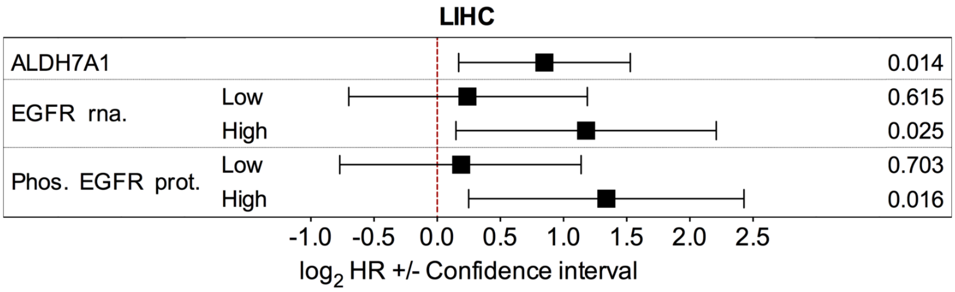

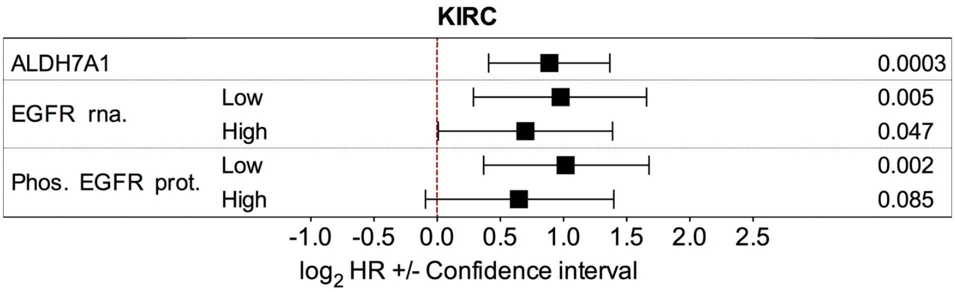

(C) Cox proportional hazard regression analysis of the association between ALDH7A1 mRNA and EGFR levels for liver and kidney cancer. The box and horizontal lines represent the estimated Hazard Ratio (HR) and corresponding confidence interval. In the liver cancer dataset association between ALDH7A1 expression and poor survival outcome is dependent on EGFR status: survival was significantly worse for patients with low ALDH7A1 in a high EGFR expression/phosphorylation group while it was not significant in low EGFR group. This was not the case for the kidney cancer patients: ALDH7A1 expression was significantly associated with poor clinical outcome in both low and high EGFR groups according to EGFR RNA expression and low EGFR group according to EGFR phosphorylation levels.

Figure S3A: Gene set and pathway analysis comparing low vs high ALDH7A1 tumors

REACTOME	piano	graphite	cepa	gage	esea
abacavir transport and metabolism				↓	
Activation of ATR in response to replication stress					↓
activation of matrix metalloproteinases		↑			
Activation of RAS in B cells					↑
Activation of the pre-replicative complex					↓
alpha linolenic acid ala metabolism	↓				
Alpha-oxidation of phytanate					↓
Amino Acid conjugation					↓
assembly of collagen fibrils and other multimeric structures		↑			
Asymmetric localization of PCP proteins					↑
AUF1 (hnRNP D0) destabilizes mRNA					↑
axon guidance	↑			↑	
Beta oxidation of decanoyl-CoA to octanoyl-CoA-CoA					↓
Beta oxidation of hexanoyl-CoA to butanoyl-CoA					↓
Beta oxidation of lauroyl-CoA to decanoyl-CoA-CoA					↓
Beta oxidation of octanoyl-CoA to hexanoyl-CoA					↓
bile acid and bile salt metabolism	↓	↓		↓	
biological oxidations	↓			↓	↓
branched-chain amino acid catabolism		↓	↕	↓	↓
Butyrate Response Factor 1 (BRF1) destabilizes mRNA					↑
cell_cycle				↑	
cell_cycle_mitotic				↑	
Chromatin modifying enzymes					↑
Chromatin organization					↑
citric_acid_cycle_tca_cycle				↓	
Clathrin derived vesicle bu↕ing					↑
collagen degradation		↑			
collagen formation		↑		↑	
Conjugation of benzoate with glycine					↓
Conjugation of carboxylic acids					↓
Conjugation of salicylate with glycine					↓
Cross-presentation of particulate exogenous antigens (phagosomes)					↓
Cross-presentation of soluble exogenous antigens (endosomes)					↑
cytochrome_p450_arranged_by_substrate_type				↓	
Deadenylation-dependent mRNA decay					↑
Dissolution of Fibrin Clot		↓			↓
DNA Damage-Telomere Stress Induced Senescence					↑
dna_replication				↑	
Elongation arrest and recovery					↑
ER-Phagosome pathway					↑
extracellular matrix organization		↑		↑	
factors involved in megakaryocyte development_and_platelet_production				↑	
fatty acid, triacylglycerol, and ketone body metabolism	↓	↑		↓	
FCGR activation					↓
Formation of ATP by chemiosmotic co↑ling					↑
formation of fibrin clot (clotting cascade)		↓		↓	
Formation of HIV elongation complex in the absence of HIV Tat					↑
Formation of HIV-1 elongation complex containing HIV-1 Tat					↑
Formation of RNA Pol II elongation complex					↑
formation of the beta-catenin-TCF transactivating complex					↑
g_alpha_i_signalling_events				↑	
G2-M Checkpoints					↓
Generation of second messenger molecules					↓
Generic Transcription Pathway					↑
gluconeogenesis				↓	
glucose metabolism	↓			↓	
glucuronidation	↓			↓	
glutathione_conjugation				↓	
glycosaminoglycan_metabolism				↑	
Golgi Associated Vesicle Biogenesis					↑
gpcr_↓stream_signaling				↑	
HATs acetylate histones					↑
HIV Transcription Elongation					↑
HIV Transcription Initiation					↑
intrinsic pathway			↕	↓	
intrinsic pathway of fibrin clot formation		↓			
L1CAM interactions					↑
lipid and lipoprotein metabolism		↓			
lipid_digestion_mobilization_and_transport				↓	
lipoprotein metabolism		↓		↓	
Meiotic synapsis					↑
metabolism of amino acids and derivatives	↓			↓	
metabolism of angiotensinogen to angiotensins		↓			
metabolism of lipids and lipoproteins	↓	↓		↓	
mitochondrial fatty acid beta oxidation	↓			↓	
Mitochondrial Fatty Acid Beta-Oxidation					↓
mitochondrial fatty acid beta-oxidation of unsaturated fatty acids					↓
mitotic_m_m_g1_phases				↑	
mitotic_prometaphase				↑	
MyD88 cascade initiated on plasma membrane					↑
ncam_signaling_for_neurite_out_growth				↑	
ncam1_interactions				↑	
Negative epigenetic regulation of rRNA expression					↑
NoRC negatively regulates rRNA expression					↑
nuclear_receptor_transcription_pathway				↓	
o-linked glycosylation		↑			
o-linked glycosylation of mucins		↑			
passive_transport_by_aquaporins				↓	
Pausing and recovery of HIV elongation					↑
Pausing and recovery of Tat-mediated HIV elongation					↑
PD-1 signaling					↓
peroxisomal lipid metabolism	↓			↓	↓
Phase 1 - Functionalization of compounds					↓
phase II conjugation	↓			↓	↓
phase1_functionalization_of_compounds				↓	
phenylalanine and tyrosine catabolism			↕		
Phosphorylation of CD3 and TCR zeta chains					↓
Platelet degranulation					↓
ppara_activates_gene_expression				↓	
PRC2 methylates histones and DNA					↑
Pyrimidine catabolism					↓
pyrimidine_catabolism				↓	
pyruvate metabolism and citric acid tca cycle				↓	
recycling of bile acids and salts		↓		↓	
Regulation of beta-cell development					↓
regulation of gene expression in beta cells	↓			↓	↓
respiratory_electron_transport				↓	
respiratory electron transport atp synthesis by chemiosmotic coupling and heat production by uncoupling proteins				↓	
Response to elevated platelet cytosolic Ca2+					↓
RNA Polymerase II HIV Promoter Escape					↑
RNA Polymerase II Promoter Escape					↑
RNA Polymerase II Transcription Elongation					↑
RNA Polymerase II Transcription Initiation					↑
RNA Polymerase II Transcription Initiation And Promoter Clearance					↑
RNA Polymerase II Transcription Pre-Initiation And Promoter Opening					↑
RORA activates circadian gene expression					↓
scavenging by class a receptors		↓			
sema3a pak dependent axon repulsion		↑			
semaphorin interactions		↑			
signaling_by_gpcr				↑	
signaling_by_ils				↑	
signaling by robo receptor				↑	
synthesis of bile acids and bile salts			↕	↓	
synthesis of bile acids and bile salts via 24 hydroxycholesterol			↕	↓	
synthesis of bile acids and bile salts via 7alpha hydroxycholesterol	↓		↕	↓	
Synthesis of PIPs at the late endosome membrane					↑
Tat-mediated elongation of the HIV-1 transcript					↑
Tat-mediated HIV elongation arrest and recovery					↑
tca_cycle_and_respiratory_electron_transport				↓	
Toll Like Receptor 10 (TLR10) Cascade					↑

REACTOME - continuation	piano	graphite	cepa	gage	esea
Toll Like Receptor 5 (TLR5) Cascade					↑
TRAF6 Mediated Induction of proinflammatory cytokines					↑
trans-Golgi Network Vesicle Bu↕ing					↑
transport of glucose and other sugars bile salts and organic acids metal ions an[illegible] amine compounds	↓				
Tristetraprolin (TTP) destabilizes mRNA					↑
U12 Dependent Splicing					↑
Unwinding of DNA					↓
Xenobiotics					↓
Zinc transporters					↑

KEGG	piano	spia	cepa	gage	esea
Adrenergic signaling in cardiomyocytes					↑
Alanine, aspartate and glutamate metabolism	↓		↕	↓	↓
Alcoholism					↑
Arginine and proline metabolism	↓		↕	↓	↓
Ascorbate and aldarate metabolism	↓			↓	
axon guidance		↑		↑	
beta-Alanine metabolism	↓		↕	↓	↓
Bile secretion				↓	
Bla↕er cancer					↑
Butanoate metabolism	↓		↕	↓	↓
Cell cycle	↑			↑	
Chemical carcinogenesis					↓
Cholinergic synapse					
Circadian entrainment					↑
Citrate cycle (TCA cycle)	↓			↓	↓
complement and coagulation cascades		↓	↕		
Cysteine and methionine metabolism					
Drug metabolism - cytochrome P450	↓			↓	↓
Drug metabolism - other enzymes	↓			↓	↓
ECM-receptor interaction		↑		↑	
Fatty acid degradation					↓
Fatty acid metabolism	↓		↕	↓	
Fc gamma R-mediated phagocytosis				↑	
Focal adhesion		↑		↑	
Folate biosynthesis				↓	
Fructose and mannose metabolism					↓
Glutathione metabolism				↓	
Glycine, serine and threonine metabolism	↓		↕	↓	↓
Glycolysis - Gluconeogenesis					↓
Glycolysis / Gluconeogenesis	↓			↓	
Glyoxylate and dicarboxylate metabolism				↓	↓
hippo signaling pathway		↑			
Histidine metabolism	↓			↓	↓
limonene_and_pinene_degradation	↓				
Lysine degradation	↓		↕		↓
MAPK signaling pathway				↑	
Maturity onset diabetes of the young					↓
maturity_onset_diabetes_of_the_young	↓				
Melanoma					↑
Metabolism of xenobiotics by cytochrome P450	↓			↓	↓
micrornas in cancer		↓			
Morphine a↕iction					↑
neuroactive ligand-receptor interaction		↓			
Osteoclast differentiation					↓
Oxidative phosphorylation				↓	
Pathogenic Escherichia coli infection					↓
pathways_in_cancer	↑				
Pentose and glucuronate interconversions	↓			↓	↓
Peroxisome	↓			↓	
Phenylalanine metabolism				↓	↓
Phenylalanine, tyrosine and tryptophan biosynthesis					↓
pi3k-akt signaling pathway		↑			
Porphyrin and chlorophyll metabolism	↓			↓	↓
PPAR signaling pathway	↓	↓		↓	
Primary bile acid biosynthesis			↕	↓	
Propanoate metabolism	↓		↕	↓	↓
Proteoglycans in cancer					↑
proximal_tubule_bicarbonate_reclamation	↓				
Pyruvate metabolism	↓			↓	↓
Regulation of actin cytoskeleton				↑	
Renin-angiotensin system				↓	
Retinol metabolism	↓			↓	↓
small cell lung cancer		↑			
Staphylococcus aureus infection					↓
Starch and sucrose metabolism	↓			↓	↓
Steroid hormone biosynthesis	↓			↓	↓
Systemic l↑us erythematosus					↓
Tryptophan metabolism	↓			↓	↓
Tyrosine metabolism				↓	
Valine, leucine and isoleucine degradation	↓		↕	↓	↓
Wnt signaling pathway				↑	

NCI	graphite	cepa	esea
Beta1 integrin cell surface interactions			↓
Beta2 integrin cell surface interactions			↓
foxa2 and foxa3 transcription factor networks	↓		↓
hnf3bpathway		↕	
integrins in angiogenesis	↑		
Regulation of nuclear beta catenin signaling and target gene transcription			↑
syndecan-1-mediated signaling events	↑		
TCR signaling in na<U+00EF>ve CD4+ T cells			↓
Urokinase-type plasminogen activator (↑A) and ↑AR-mediated signaling			↓

BIOCARTA	cepa	gage	esea
reckpathway	↕		
intrinsic pathway		↓	
the information processing pathway at the ifn beta enhancer			↑
intrinsic prothrombin activation pathway			↓
extrinsic prothrombin activation pathway			↓
basic mechanism of action of ppara pparb(d) and pparg and effects on gene expression			↓

PANTHERA	graphite	esea
Integrin signalling pathway	↑	
Transcription regulation by bZIP transcription factor		↑
General transcription regulation		↑
Plasminogen activating cascade		↓

HALLMARK GENE SETS (MSigDB Collection)	piano	gage
adipogenesis	↓	↓
allograft_rejection	↑	↑
angiogenesis		↑
apical_junction		↑
bile acid metabolism	↓	↓
e2f_targets		↑
epithelial mesenchymal transition	↑	↑
fatty_acid_metabolism	↓	↓
g2m_checkpoint	↑	↑
heme_metabolism		↓
inflammatory_response		↑
mitotic_spindle		↑
oxidative_phosphorylation	↓	↓
peroxisome	↓	↓
tnfa_signaling_via_nfkb	↑	↑
xenobiotic_metabolism	↓	↓

(A) For each method and corresponding annotation set, significantly affected pathways and biological processes where selected (p<0.05). Pathways and biological processes that were changed in both LIHC and KIRC patients with low ALDH7A1 are shown. ↑ - activated; ↓- inactivated pathways and biological processes; ↕- direction is not provided.

S3B: Effects of low ALDH7A1 on pathways in KIRC

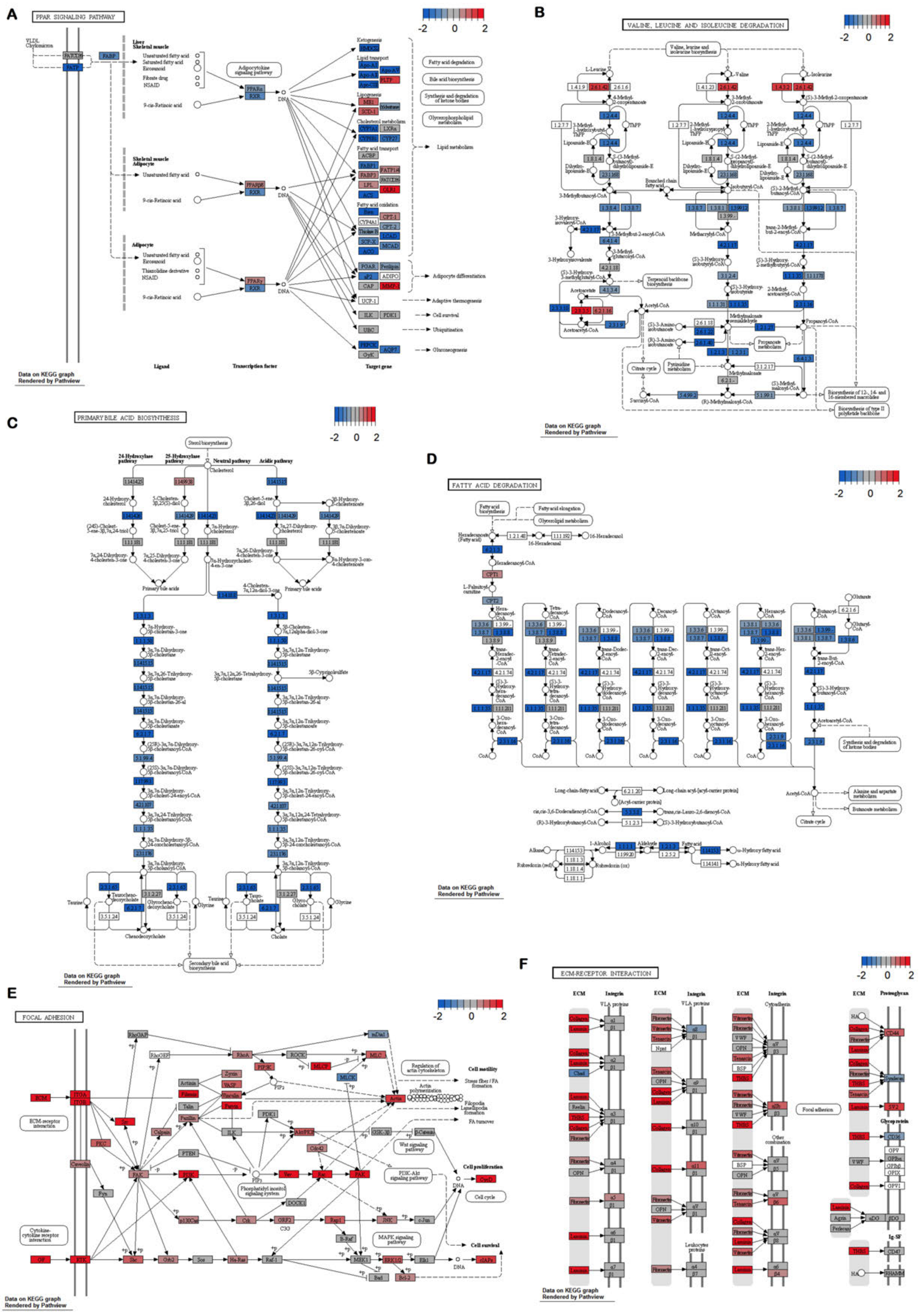

Figure S4: metabolite profiles on cancer cell lines

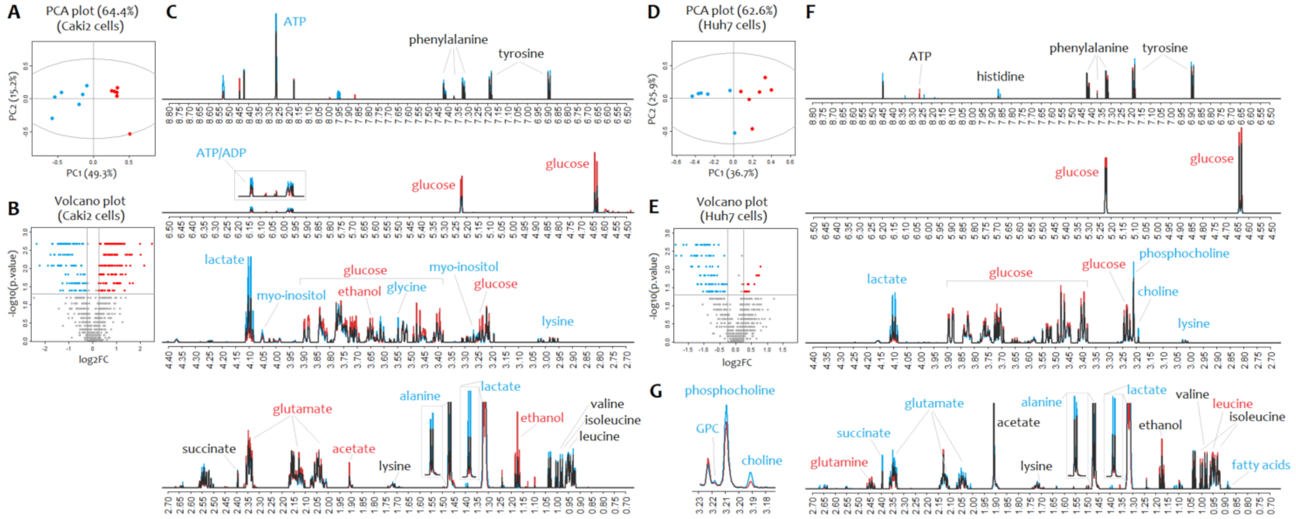

(A, D) Principal component analysis shows a clear separation between control and ALDH7A1 depleted cells (Blue dots – control cells, red dots – ALDH7A1 depleted cells). This difference is somewhat smaller in magnitude than in BJ cells.
(B, E) Volcano plot of significance versus $\log_2$ fold change of all intensity points of the spectra above median. The x axis – $\log_2$FC between control and ALDH7A1 depleted cells (threshold $\log_2$FC +/- 0.25). The y axis - significantly increased (red) or decreased (blue) points (p. value >0.05).
(C, F) Average ^{1}H NMR spectra of control (blue) and ALDH7A1 depleted cells (red). Red - significantly upregulated metabolites; blue - downregulated metabolites; black – no change.
(G) Zoomed in region of spectra (3.23-3.18) where phosphocholine and glycerophosphocholine peaks are located.

In all three cell lines, lactose levels decreased and glucose levels increased. In Huh7 cells we see a reduction in glycerophosphocholine (GPC), phoshocholine and choline levels. We were not able to detected phosphocholine and glycerophosphocholine (GPC) in caki2 cells as it was below the detection level. The effects on amino acids were cell line dependent.

Huh7 and Caki2 ^{1}H NMR spectra were processed and analyzed as described for BJ cells in the methods section. Briefly spectra were normalized against total intensity of a spectral region (above 1.5). “CluPA” algorithm was used to align peaks. “Rolling ball” algorithm (span – 50) was applied to correct shifting baseline. Baseline correction, data binning (bin=4), normalization and peak alignment was done using R package “ChemoSpec”.

Figure S5: assessment of correlation between PPAR activity and ALDH7A! on other cancers

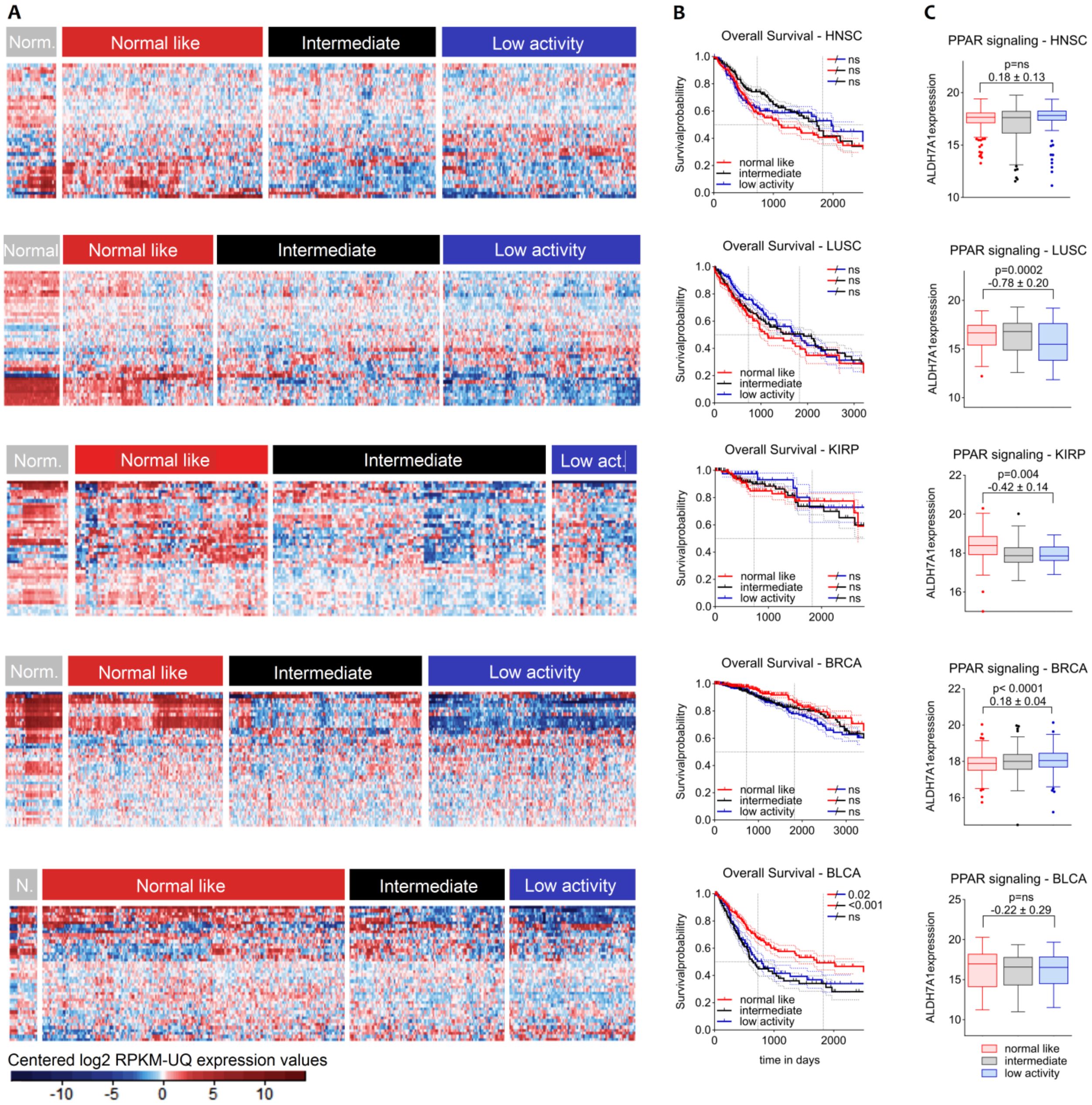

(A) The “low activity”, “intermediate” and “normal like” PPAR signature groups were identified as described for Figure 5.

(B) Survival outcome was compared as described in Figure 5. The HNSC, LUSC, KIRP, BRCA patient groups with low PPAR activity did not exhibit significantly lower overall survival probability compared to “normal-like” PPAR group for these cancers. BLCA showed worse survival for both the low and intermediate PPAR groups compared to the normal-like group.

(C) ALDH7A1 expression was assessed in the three PPAR activity groups, as described in figure 5. There was no correlation between low PPAR activity and low ALDH7A1 levels.

Figure S6: effects of PPAR agonists

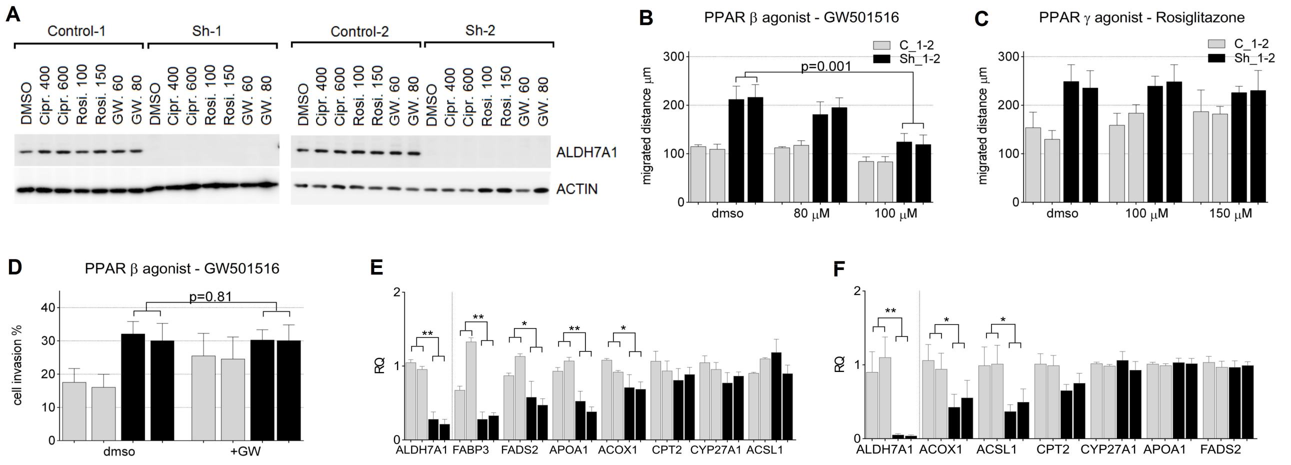

(A) Immunoblots showing ALDH7A1 protein in BJ-4F3 cells treated with the PPAR agonists. The PPARα agonist ciprofibrate (Cipr) was used at 400 and 600 μM; the PPARβ agonist GW501516 (GW) was used at 60 and 80 μM; the PPARγ agonist rosiglitazone (Rosi) was used at 100 μM and 150 μM. Sh-1 and sh-2 show the effect of shRNA mediated depletion of ALDH7A1. Control 1 (C-1) indicates cells transduced with the empty vector. Control 2 (C-2) expressed a non-targeting shRNA Anti-actin was used to control for loading.

(B-C) Quantification of wound healing assays after 24h migration. Cells were treated with PPARβ and PPARγ agonist or DMSO as a control. The migrated distance was measured (μm), and averages from three independently transduced cell lines were calculated (± SEM)

(D) Quantification of cell invasion through Matrigel over 24h. BJ-4F3 cells were treated with PPARβ agonist or DMSO as a control. The bar plots show the percent of cells that crossed the matrigel barrier (average of 3 independent experiments ± SEM). The two-tailed Mann Whitney test was used to calculate p-values.

(E-F) RT-PCR of PPAR transcriptional targets. Light grey – control cells transduced with the empty vector and non-targeting shRNA, accordingly. Black – ALDH7A1 depleted cells transduced with two independent shRNAs (sh-1 and sh-2). Data represent average ± standard error of the mean (SEM) from 3 independent experiments normalized to β-actin, kif1 and tbp (in the case of Huh7 cells) and kif1 (Caki2 cells). The two-tailed Mann Whitney test with adjustment for False Discovery Rate was used to calculate p-values.

(G) RT-qPCR of PPAR transcriptional targets in cells treated with Ciprofibrate. Light grey – control cells transduced with non-targeting shRNA and ALDH7A1 depleted cells transduced with shRNAs (sh-1). Cells were seeded and allowed to attach overnight and then treated with Ciprofibrate or DMSO. Cells were collected for RNA extraction and RT-qPCR. β-actin was used as normalization control. Friedman rank sum test with pairwise post-hoc test for multiple comparisons with holms adjustment was used to calculate p-values between groups with and without Ciprofibrate treatment.

Data represents average ± SEM from 2 independent experiments.

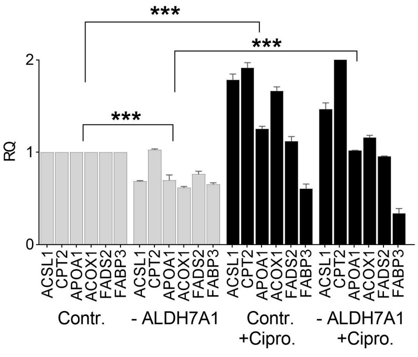

Figure S7: Assays on cancer cell lines

Cell line	Tissue	Migration [1]	PPAR [2]	Rescue [3]	Source	Mycoplasma tested	validation
HepG2	Liver	Yes on collagen	No	Yes	Hong lab IMCB A*STAR, 2012	Hong Lab	phenotype, behaviour
HepG2	Liver	Yes on collagen	No	Yes	Bisgaard lab, ICMM, KU, 2016	Bisgaard lab	phenotype, behaviour
C3A	Liver	Yes on collagen	No	Yes	ATCC, product CRL-10741, 2017	ATCC	ATCC
HepRC	Liver	No	not done	not done	Bisgaard lab, ICMM, KU, 2016	Bisgaard lab	Bisgaard lab
Hep3B	Liver	not done [4]	not done	not done	Hong lab IMCB A*STAR, 2012	Hong Lab	phenotype, behaviour
JHH6	Liver	No	No	not done	JCRB Cell Bank, JCRB1030, 2017	JCRB	JCRB
JHH7	Liver	growth arrest	not done	not done	JCRB Cell Bank, JCRB1031, 2017	JCRB	JCRB
HUH7	Liver	Yes	Yes	Yes	Bisgaard lab, ICMM, KU, 2016	Bisgaard lab	phenotype, behaviour
BFTC-909	Kidney [4]	No	No	not done	DMSZ, ACC 367, 2017	DMSZ	DMSZ
Caki2	Kidney	Yes	Yes	Yes	Bisgaard lab, ICMM, KU, 2016	Bisgaard lab	Bisgaard lab
Caki1	Kidney	growth arrest	not done	not done	Bisgaard lab, ICMM, KU, 2016	Bisgaard lab	Bisgaard lab

Notes:

1) Scratch assays were performed as described in Figure 1.

2) “PPAR signature” was examined by qPCR for selected PPAR targets, as in Figure 5. Yes indicates changes in PPAR target expression.

3) “Rescue” indicates suppression of the cellular phenotype by treatment with the PPAR alpha agonist, as in Figure 6.

4) Hep3B cells detach very easily, so the scratch assay cannot be done. All other kidney cell lines we tested do not grow as a monolayer that lends itself to this kind of assay.

Figure S8. Clinical characteristics of the patients included in the study

LIHC (n=120)

Stage	Nr. of patients
I	7
II	54
III	56
IV/III-IV	3
**Grade**	**Nr. of patients**
G1	4
G2/G1-G2	75
G3/G2-G3	41
**STATUS**	**Nr. of patients**
Survivors	54
Deceased	66
**Follow up time**	
Average, SD	29.1 +/- 17.9
Median	26.5
Media among the survivors	39
Median among the deceased	17.5
**ALDH7A1 depletion score**	**Nr. of patients**
-4	8
-3	14
-2	23
-1	30
0	37
1	6
2	1
3	1
**ALDH7A1 score (Normal tissue)**	**Nr. of patients**
4	66
3	32
2	13
1	7
0	1
**ALDH7A1 score (Tumor tissue)**	**Nr. of patients**
4	27
3	24
2	24
1	28
0	17

KIRC (n=149)

Stage	Nr. of patients
I	75
II	24
III	27
IV/III-IV	19
**Grade**	**Nr. of patients**
G1	29
G2/G1-G2	41
G3/G2-G3	50
G4/G3-G4	20
**STATUS**	**Nr. of patients**
Survivors	89
Deceased	59
**Follow up time**	
Average, SD	57.4 +/- 24.4
Median	68
Media among the survivors	74
Median among the deceased	34
**ALDH7A1 depletion score**	**Nr. of patients**
-4	2
-3	5
-2	21
-1	40
0	57
1	23
**ALDH7A1 score (Normal tissue)**	**Nr. of patients**
4	72
3	60
2	14
1	2
**ALDH7A1 score (Tumor tissue)**	**Nr. of patients**
4	41
3	56
2	36
1	12
0	3
